# Toward Robust ARB Inactivation in Wastewater: Comparing Performic Acid, Peracetic Acid, and Chloramination Using ICT, Resistance, and Reactivation Metrics

**DOI:** 10.1002/wer.70461

**Published:** 2026-07-05

**Authors:** Nuha Alfahham, Katherine Y. Bell, Reem Suleiman, John Norton, Glen T. Daigger

**Affiliations:** ^1^ Department of Civil and Environmental Engineering University of Michigan Ann Arbor Michigan USA; ^2^ Brown and Caldwell Troy Michigan USA; ^3^ Hazen and Sawyer Nashville Tennessee USA; ^4^ Department of Biomedical Engineering University of Michigan Ann Arbor Michigan USA; ^5^ Great Lakes Water Authority Detroit Michigan USA

**Keywords:** antimicrobial resistance, integrated concentration–time, peracetic acid, performic acid, sodium hypochlorite, vancomycin‐resistant enterococci, viable but nonculturable bacteria, wastewater disinfection

## Abstract

Antimicrobial resistance (AMR), particularly vancomycin‐resistant enterococci (VRE), poses a critical global health threat. Although wastewater disinfection limits pathogen dissemination, conventional chlorine‐based disinfection can be ineffective against antibiotic‐resistant bacteria (ARB) and may induce viable but nonculturable (VBNC) states. This study compared performic acid (PFA), peracetic acid (PAA), and sodium hypochlorite (NaOCl) for inactivation of VRE and total enterococci (TE) in high‐ammonia secondary effluent. Batch disinfection experiments were conducted using an integrated concentration–time (ICT) framework, assessing microbial survival under standard (24 h) and extended (3–5 days) incubation, to quantify posttreatment recoverability (reactivation/regrowth). PFA achieved rapid and consistent inactivation within the effective linear range, whereas PAA exhibited slower, variable kinetics with distinctive shouldering. NaOCl proved the least reliable and displayed a “lower dose advantage.” Reactivation was strongly dependent on inactivation magnitude. At the doses investigated, only PFA consistently achieved > 2.5‐log reductions associated with minimal recovery. By comparison, PAA and NaOCl did not reach inactivation levels adequate to suppress reactivation within the tested ICT ranges, indicating VBNC states and regrowth. Within the tested ICT ranges in GLWA secondary effluent, these findings suggest PFA is a promising alternative disinfectant for controlling ARB and provide a reactivation‐aware framework for setting exposure targets based on durable, not just immediate, inactivation.

## Summary

- Performic acid (PFA) provided consistent, rapid inactivation of total enterococci and vancomycin‐resistant enterococci in high‐ammonia secondary effluent.
- Effective disinfection may require exceeding an integrated concentration–time (ICT) threshold of approximately 28 mg·min/L to minimize posttreatment microbial recovery.
- Peracetic acid and sodium hypochlorite exhibited variable inactivation and substantial reactivation under tested conditions, consistent with VBNC injury and/or regrowth of persistent antibiotic‐resistant bacteria (ARB).
- Incorporating postdisinfection reactivation into ICT‐based evaluation provides frameworks for exposure targets and supports risk‐informed selection of disinfectants for ARB control.
- PFA typically adds less COD than PAA and may avoid neutralization but requires onsite generation.

## Graphical abstract

Performic acid (PFA) achieved rapid, linear, and consistent inactivation of enterococci and vancomycin‐resistant enterococci, peracetic acid (PAA) exhibited slower, variable kinetics with distinctive shouldering, and sodium hypochlorite proved the least reliable and displayed a “lower dose advantage.” These findings position PFA as a promising alternative disinfectant for controlling antibiotic‐resistant bacteria.

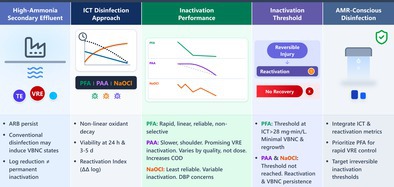

**Abbreviations**
AA: acetic acid
ACS: American Chemical Society
AMR: antimicrobial resistance
ANCOVA: analysis of covariance
AOP: advance oxidation process
ARB: antibiotic‐resistant bacteria
ARG(s): antibiotic resistance gene(s)
ATP: adenosine triphosphate
BOD: biochemical oxygen demand
BSL‐1: Biosafety Level 1
CDC: Centers for Disease Control and Prevention
CFU: colony‐forming units
COD: chemical oxygen demand
CT: concentration × time (disinfection surrogate)
DBP(s): disinfection byproduct(s)
DI: deionized water
DNA: deoxyribonucleic acid
DPD: N,N‐diethyl‐p‐phenylenediamine (colorimetric method for chlorine/chloramine)
EPA: United States Environmental Protection Agency
FA: formic acid
FC: free chlorine
GLWA: Great Lakes Water Authority
HGT: horizontal gene transfer
HOCl: hypochlorous acid
ICT: integrated concentration–time (time‐resolved exposure)
IDEXX: IDEXX Laboratories (commercial system used for Enterolert/Quanti‐Tray enumeration)
LOD: limit of detection
MGD: million gallons per day
MPN: most probable number (IDEXX enumeration output)
NA: nucleic acid
NaOCl: sodium hypochlorite
OC: organic chloramines
OLS: ordinary least squares (regression)
OP(s): opportunistic pathogen(s)
PAA: peracetic acid
PFA: performic acid
QMRA: Quantitative Microbial Risk Assessment
RI: reactivation index (ΔΔlog; extended vs. standard incubation)
S: slope
TC: total chlorine (preferred oxidant metric in ammonia‐rich matrices)
TE: total enterococci
TSS: total suspended solids
UV: ultraviolet (disinfection)
VBNC: viable but nonculturable
VRE: vancomycin‐resistant enterococci
WHO: World Health Organization
WRF: Water Research Foundation (project reference “WRF 5219”)
WRRF: Water Resource Recovery Facility (used interchangeably with WWTP)
WWTP: wastewater treatment plant

## Symbols, Units, and Notational Conventions

**Table:** 

Symbol/unit	Meaning	Notes
mg·min·L⁻¹	ICT unit	Integrated oxidant exposure
mg/L	Concentration	Disinfectant dose; COD/TSS/TDS measures
μS/cm	Conductivity	Bulk water parameter
CFU/100 mL	Enumeration	IDEXX/MPN reporting
*R* ^2^	Coefficient of determination	Regression fits for kinetics
Δlog (LR)	Log_10_ reduction	Primary inactivation endpoint
ΔΔlog	Reactivation index	Extended—Standard incubation difference
*p*‐value	Statistical significance	Thresholds reported (e.g., < 0.05)

## 1 Introduction

With nearly 5 million global deaths linked to antibiotic‐resistant bacteria (ARB) in 2019 alone and projections forecasting 10 million annual fatalities by 2050, the societal and financial burden is staggering, risking an annual GDP shortfall of US $3.4 trillion and pushing millions into extreme poverty (Franklin et al. 2024). Among these critical pathogens, vancomycin‐resistant enterococci (VRE) are particularly concerning, with a worldwide distribution spanning clinical, community, and environmental niches (Almeida‐Santos et al. 2025). *Enterococci* species are naturally occurring commensals in the gastrointestinal tracts of humans and animals, but they can become opportunistic pathogens (OPs). Their capacity to accumulate and disseminate resistance genes makes them reservoirs of antimicrobial resistance (AMR) determinants (Almeida‐Santos et al. 2025). As prevalent nosocomial pathogens responsible for serious infections, including bloodstream infections with high morbidity and mortality, VRE are often difficult to treat due to their remarkable ability to survive in harsh environments; they form biofilms and readily transfer resistance genes such as *vanA* and *vanB*, allowing VRE to act as reservoirs and vectors for AMR determinants (Zhou et al. 2020). Vancomycin is often regarded as a last‐resort antibiotic for severe Gram‐positive infections; the emergence of resistance directly compromises one of the few remaining effective therapies (Li et al. 2022).

Because of their resistance to disinfection and association with worldwide infections, VRE are classified as a high priority by the World Health Organization (WHO) on its global priority pathogens list for research and development of new antibiotics (Almeida‐Santos et al. 2025; Sati et al. 2025). Additionally, the United States (US) Centers for Disease Control (CDC) and Prevention identified VRE as a serious public health threat, with over 54,000 VRE‐related hospitalizations and more than 5000 deaths annually in the United States, leading to significant healthcare costs (CDC 2019).

The United Nations Environment Assembly acknowledged the environmental importance of AMR, emphasizing the critical role of wastewater treatment plants (WWTPs) in curbing its spread (Wernli et al. 2022). WWTPs receive a complex mixture of microorganisms, antibiotics, and other constituents from hospitals, households, and industries (Abia et al. 2023). Although untreated wastewater serves as a major hotspot for the evolution and dissemination of AMR, WWTPs present an opportunity to intercept, reduce, and potentially eliminate these resistance factors before they re‐enter natural environments. The rising presence of ARB and antibiotic resistance genes (ARGs) in aquatic ecosystems, and the presence of VRE in wastewaters and rivers, have been documented (Łuczkiewicz et al. 2010). Notably, hospital‐associated clones of VRE have been detected in municipal WWTP effluents, suggesting that treatment processes may not prevent downstream environmental contamination, underscoring the need for more effective strategies to control AMR. A comparison of secondary wastewater treatment technologies for ARB and ARGs removal in Michigan, US, indicated that conventional treatment processes, including activated sludge, oxidation ditches, and rotating biological contactors, were significantly less effective than membrane bioreactors (Munir et al. 2011).

The effectiveness of wastewater disinfection is highly dependent on the mechanism of disinfectant action and the specific microbial targets. Despite their role in reducing fecal indicators, conventional chlorine‐based disinfection methods can fail to neutralize resistant strains, particularly VRE, and can even facilitate their positive selection; many disinfection processes can induce incomplete inactivation and/or a viable but nonculturable (VBNC) state rather than irreversible destruction, allowing microbial reactivation and persistence (Mosaka et al. 2023). The efficacy of disinfection is further complicated by the intrinsic resistance of Gram‐positive bacteria, like enterococci, to chemical oxidation, often leading to differential responses compared with Gram‐negative indicators. Given the clinical implications of VRE colonization, including elevated mortality rates, prolonged hospital stays, and increased healthcare costs, innovative VRE control measures are important for reducing the burden on healthcare systems and preventing further spread of multidrug resistance (Almeida‐Santos et al. 2025).

Chlorination (or chloramination in the presence of relatively high ammonia concentrations) has been widely used but is associated with the formation of harmful disinfection byproducts (DBPs) and potential selection for AMR (Umar 2022). Free chlorine (FC) acts as a potent oxidant that rapidly damages cell membranes, proteins, and enzymes through electrophilic attack. In contrast, monochloramine (NH_2_Cl), the dominant residual species when chlorine is applied in unnitrified secondary effluents, acts as a slower‐penetrating biocide that oxidizes intracellular sulfhydryl groups and damages nucleic acids (NAs), reducing bacterial viability (Gray et al. 2013). In chlorinated effluents, ARB and VBNC cells can persist and reactivate, retaining their potential for virulence and horizontal gene transfer (HGT). Moreover, vancomycin resistance genes, such as *vanA* and *vanB*, can remain detectable in chlorinated effluents, despite declines in culturable VRE numbers (Furukawa et al. 2015). These challenges necessitate exploration and comparative assessment of alternative disinfection chemistries, accounting for their unique inactivation mechanisms and impact on microbial viability.

Peracetic acid (PAA) and performic acid (PFA) (Equations 1 and 2), which offer broad‐spectrum antimicrobial activity and improved environmental safety (Bagagnan et al. 2024), emerged as alternative disinfectants. These compounds are synthesized by combining a weak organic acid with hydrogen peroxide, producing a strong oxidizer capable of damaging cell membranes and genetic material (Bagagnan et al. 2024).

PAA Formation:

(1) $${\mathrm{CH}}_{3}{\mathrm{CO}}_{2}\;H+H_{2}O_{2}⇌{\mathrm{CH}}_{3}{\mathrm{CO}}_{3}\;H+H_{2}O$$

where

- CH_3_CO_2_ H = acetic acid (AA)
- H_2_O_2_ = hydrogen peroxide
- CH_3_CO_3_ H = PAA
- H_2_O = water

PFA Formation:

(2) $$\text{HCOOH}+H_{2}O_{2}⇌{\mathrm{CH}}_{2}O_{3}+H_{2}O$$

where

- HCOOH = formic acid (FA)
- H_2_O_2_ = hydrogen peroxide
- CH_2_O_3_ = PFA
- H_2_O = water

Although both are peracids, their molecular reactivity and specific modes of action differ. PFA is a highly potent oxidant that reacts rapidly with cellular constituents, causing extensive and immediate damage (Ding et al. 2023). In mechanistic analyses of PFA disinfection, inactivation via both direct (driven primarily by the parent peroxyacid molecule) and indirect (mediated by reactive radicals) reactions has been observed; under tested wastewater conditions, direct reactions of the PFA molecule dominated, whereas indirect contributions were attributed to •OH and other peroxide‐radical pathways (Ding et al. 2023). In contrast, PAA typically shows lower efficacy than PFA in wastewater, and its observed performance can be more sensitive to matrix effects. Across peroxyacids, reported mechanistic evidence indicates selective oxygen‐transfer reactivity toward reduced‐sulfur targets (e.g., cysteine and methionine) and intracellular oxidative stress following membrane penetration, rather than nonspecific attack (J. Wang et al. 2023).

This Gram‐positive relevance is critical for VRE (Gram‐positive enterococci), which can require greater exposure to achieve comparable log reductions relative to Gram‐negative indicators under similar wastewater conditions (Ragazzo et al. 2020).

Because oxidative disinfection can cause sublethal injury, an initial loss of culturability may not necessarily indicate irreversible inactivation, and recovery may occur during subsequent incubation; accordingly, viability assessment via extended incubation provides an operational check for resuscitation of injured/VBNC cells following disinfection (Antonelli et al. 2013; Ding et al. 2023).

Studies indicate that peracid‐based disinfectants may effectively reduce the percentages of ARB (Ragazzo et al. 2020). Importantly, indirect (radical‐mediated) pathways are generally considered nonselective with respect to antibiotic‐resistance phenotypes because highly reactive radicals (especially •OH) attack diverse biomolecular targets rather than antibiotic‐specific pathways; consistent with this, ozone‐based advanced oxidation process (AOP) achieved > 99.9% inactivation of antimicrobial‐resistant bacteria, including VRE, without significant differences relative to antimicrobial‐susceptible bacteria under the same treatment conditions (Azuma et al. 2023). Additionally, these agents decompose into byproducts such as water, oxygen, and AA or FA, making them appealing for environmentally sustainable disinfection (Usman et al. 2023).

PAA has broad antimicrobial activity and can effectively reduce ARB in wastewater effluents (Ragazzo et al. 2020). However, its disinfection performance may vary depending on factors such as pH, organic matter content, and contact time. In some cases, sublethal oxidative stress caused by PAA may not completely inactivate bacteria, potentially allowing resistant subpopulations or VBNC cells to persist and later reactivate (Antonelli et al. 2013).

PFA has emerged more recently as a promising alternative, with rapid disinfection efficacy at low concentrations and minimal environmental residue (Gehr et al. 2009). Nonetheless, comprehensive comparative research on the effectiveness of PAA and PFA, particularly against specific ARB groups like VRE and in direct comparison with conventional disinfectants like chlorine (or chloramines), remains important for understanding their full impact on mitigating ARB spread and long‐term microbial control (Almeida‐Santos et al. 2025). The lack of data on PFA effectiveness against VRE presents a critical knowledge gap, especially as these organisms pose threats in both clinical and environmental contexts (Almeida‐Santos et al. 2025). Addressing this research gap will aid in advancing disinfection strategies that could mitigate ARB spread throughout treated wastewater effluents (Franklin et al. 2024).

This study assesses the disinfection efficacy of PFA compared with PAA and NaOCl. Specific objectives include comparing the inactivation efficacy of total *enterococci* (TE) and VRE among disinfectants, and assessing the potential for sublethal injury, reactivation, and selection of ARB following disinfection. Secondary effluent from the Great Lakes Water Authority (GLWA) Water Resource Recovery Facility (WRRF) in Detroit, MI, the largest single‐site wastewater treatment facility in North America (Li et al. 2024), was used to conduct these studies. The WRRF receives and treats residential, industrial, and commercial waste, along with stormwater, depending on the various service areas (Li et al. 2024), making it an exemplary setting for evaluating advanced disinfection methods. As one of the first evaluations of PFA in the United States, and using effluent from a full‐scale WRRF, the results fill an important knowledge gap. This study is part of a broader research project (Water Research Foundation [WRF] 5219) conducted by GLWA to evaluate alternative disinfection methods.

## 2 Materials and Methods

This study evaluated the efficacy of PFA disinfection in comparison with PAA and NaOCl against both TE and VRE in unnitrified secondary effluent samples. Selecting TE and VRE was a priority because (i) enterococci are United States Environmental Protection Agency (EPA)–recognized fecal indicators for recreational/wastewater interfaces, (ii) VRE are a CDC‐designated “Serious Threat” and a common healthcare‐associated pathogen (CDC 2019), and (iii) both are compatible with standardized, field‐transferable enumeration using the IDEXX Enterolert system with vancomycin‐amended trays used in this study; this focused pairing is a pragmatic choice to provide initial data related to ARB disinfection and would be useful in informing future work with additional taxa.

### 2.1 Sample Collection and Preparation

The GLWA WRRF has a peak secondary treatment capacity of 3,520,000 m^3^/day (930 million gallons per day [MGD]) and a primary treatment capacity of 6,400,000 m^3^/day (1700 MGD) (Ko 2025). The liquid process train includes influent pumping, preliminary treatment, ferric chloride addition for phosphorus removal, primary clarification, nonnitrifying high‐purity oxygen activated sludge incorporating biological phosphorus removal for 5‐day biochemical oxygen demand (BOD), total suspended solids (TSS), and total phosphorus removal, chlorination, and dechlorination with sodium bisulfite (Gray et al. 2013). Primary and waste activated sludges are thickened separately in gravity thickener complexes. Treatment facility performance is stable, routinely meeting secondary treatment and effluent total phosphorus limits (0.6 mg P/L for the summer [April–September] and 0.7 mg P/L during the winter, October–March) (Ko 2025). Over the most recent 4‐year period, effluent BOD averaged 5.8 mg/L (± 2.9 mg/L), TSS 7.3 mg/L (± 3.2 mg/L), and ammonia‐N 11.4 mg/L (± 3.5 mg/L) (Ko 2025).

Secondary effluent samples were obtained from March to May 2025. Samples collected on March 29 and April 5 were used to develop modified microbial methods and conduct preliminary AMR assessments, respectively (Sections S1–S2). Results are presented in Figure S2 and demonstrate resistance of enterococci and *Escherichia coli* to three tested antibiotics, with vancomycin resistance of 7.5% and 52.5% to these bacteria, respectively. The AMR assessments characterized resistance profiles of prevalent resistant indicator bacteria in wastewater and verified reliable quantification of target AMR postdisinfection. Samples for the subsequent disinfection study were collected on April 13, May 3, and May 4. Effluent parameters were measured according to standard methods (Table 1). Samples (10 L each) were collected in autoclaved, high‐density polyethylene carboys (Thermo Fisher Scientific, Waltham, MA, USA; Cat. No. 2197‐0020), with aliquots (1.5 L) subsampled for chemical oxygen demand (COD), TSS, pH, and temperature measurements. TSS (1 L) and COD (250 mL) samples were immediately transferred to bottles, preserved on ice, and transferred to a temperature‐controlled room (4°C). Sample temperature and pH were measured immediately using a portable multiprobe. The remaining samples were partitioned into six 1‐L aliquots for subsequent disinfection treatments.

**TABLE 1:** Wastewater quality parameters, analytical methods, holding times, and preservation conditions used in this study.

Parameters	Method	Holding time	Preservation
COD	USEPA 410.4	28 days	Cool ≤ 6°C, H_2_SO_4_ to pH < 2
TSS	SM 2540D—2015	7 days	Cool ≤ 6°C
Temperature	Probe	Immediately	None
pH	Probe	Immediately	None

### 2.2 Chemical Disinfectants

Stock concentrations of NaOCl (reagent grade, 10%–15% available chlorine; Fisher Scientific, Catalog No. NC2081766) and PAA (32 wt.% in dilute AA; Sigma‐Aldrich Fine Chemicals Biosciences, St. Louis, MO, USA; Cat. No. 501884099) were utilized for disinfection experiments. PFA (9%–13% [w/v]) was synthesized in the laboratory following a previously established protocol (Gehr et al. 2009) with slight modifications. First, catalyzed FA was prepared by slowly adding 0.47 mL of H_2_SO_4_ (sulfuric acid; 95%–98%; Spectrum Chemical Manufacturing Corporation, New Brunswick, NJ, USA; Cat. No. 18‐607‐165) to 5 mL FA (formic acid; 88 wt%; American Chemical Society [ACS] Reagent; LabChem Inc., Zelienople, PA, USA; Cat. No. 01338176) over ice. While continuously mixing with a magnetic stirrer (Fisher Scientific, Hampton, NH, USA; Cat. No. 11676265), 5 mL of hydrogen peroxide (35‐wt% solution, stabilized; Thermo Scientific Chemicals, Waltham, MA, USA; Cat. No. AC202460250) was added to the prepared FA solution and maintained in an ice bath, and the mixture temperature was kept below 15°C. After 90 min of continuous, slow mixing, the PFA solution was used following validation of the stock solution strength. Final PFA solution concentrations ranged between 9% and 13% (w/v), determined by dilution and analysis using the CHEMetrics Peracetic Acid SAM kit (DPD, N,N‐diethyl‐p‐phenylenediamine, Colorimetric Method; CHEMetrics, Midland, VA, USA; Cat. No. I‐2020). Measured concentrations were converted to PFA by applying a factor of 0.816. Remaining PFA solutions following disinfection experiments were stored at −20°C for up to 3 weeks. Stored stock was monitored periodically, including prior to experimental use, using the same dilution protocol and analytical method to confirm consistency. Measured values remained within a narrow range with no systematic decrease over the monitoring interval, indicating no detectable loss of stock strength, whereas minor variation between measurements is attributed to routine analytical and dilution variability.

### 2.3 Experimental Disinfection Trials

**FIGURE 1:**
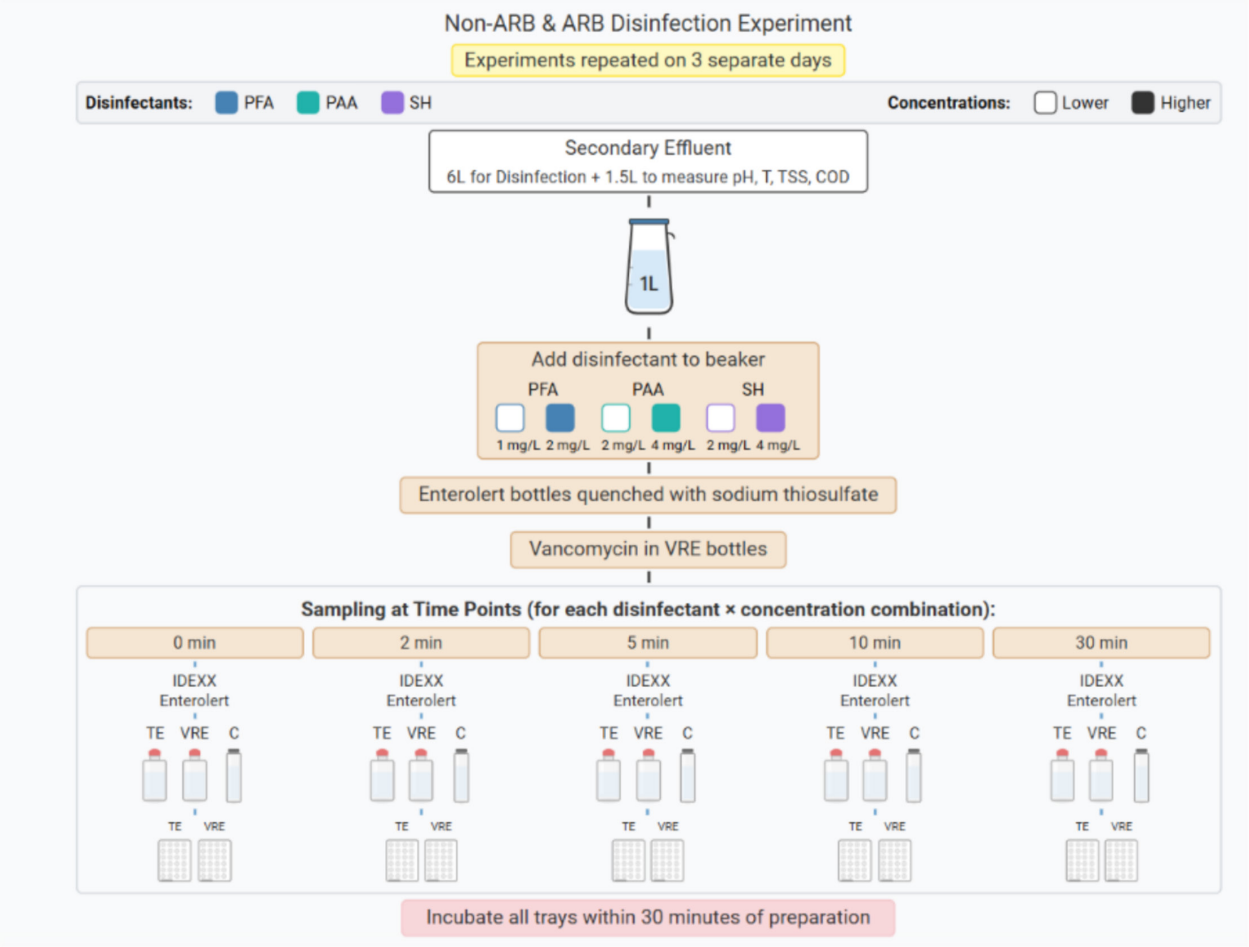
Bench‐scale disinfection workflow for total enterococci (TE) and vancomycin‐resistant enterococci (VRE) in secondary effluent, showing disinfectant dosing, time‐point sampling (0–30 min), residual measurement in unquenched aliquots, quenching, and IDEXX Enterolert analysis under standard and extended incubation.

Table 2 presents the experimental design. Six batch disinfection experiments were conducted for each of three secondary effluent samples, consisting of two different initial target disinfectant doses for each disinfectant. Four microbial analyses (TE standard and extended, and VRE standard and extended) were conducted for each batch disinfection experiment. Each experiment was conducted in a separate bench‐scale glass reactor containing 1 L of secondary effluent, as illustrated in Figure 1. Each disinfectant was dosed into a beaker separately to achieve the target initial concentration, and samples were collected at defined contact times (0, 2, 5, 10, 30 min).

**TABLE 2:** Experimental matrix of disinfectants, target doses, and microbial analyses conducted for total enterococci (TE) and VRE under standard and extended incubation conditions. An “X” indicates combinations evaluated.

Disinfectants and initial target dose	Microbial analysis			
	TE		VRE	
	Standard	Extended	Standard	Extended
Performic acid (PFA)				
1 mg/L	X	X	X	X
2 mg/L	X	X	X	X
Peracetic acid (PAA)				
2 mg/L	X	X	X	X
4 mg/L	X	X	X	X
Sodium hypochlorite (as Cl_2_)				
2 mg/L	X	X	X	X
4 mg/L	X	X	X	X

Subsamples were immediately quenched in 100‐mL IDEXX Laboratories (commercial system used for Enterolert/Quanti‐Tray enumeration) (IDEXX) sample vessels prefilled with sodium thiosulfate and bisulfate (IDEXX Laboratories, Westbrook, ME, USA; Cat. No. WV120SBST‐200) for microbial and AMR analysis. These vessels are specified to neutralize at least 15‐mg/L residual chlorine in a 100‐mL sample; the sufficiency of this capacity relative to applied oxidant concentrations was evaluated on a stoichiometric basis (Section S3.1). PAA and PFA were not detected in quenched samples. Unquenched subsamples (30 mL) were transferred into disinfectant analysis tubes for total chlorine (TC) and FC using a DR900 colorimeter (Hach Company, Loveland, CO, USA; USEPA DPD Methods 10250 and 10245, respectively); PAA and PFA were analyzed using the CHEMetrics Peracetic Acid SAM kit. A conversion factor of 0.816 was applied for PFA concentration determination to account for the difference in molecular oxidative capacity. The basis for this conversion and the disinfection experimental protocol are provided in Section S3.

### 2.4 Microbial and Antibiotic Resistance Analysis

Standard EPA‐approved Enterolert (IDEXX Laboratories, Westbrook, ME, USA; Cat. No. WENT200) test kits with the Quanti‐Tray system (IDEXX Laboratories, Westbrook, ME, USA; Cat. No. WQT‐2K) were used to enumerate TE, following established methods for wastewater monitoring (Sercu et al. 2010). The IDEXX system offers several advantages over traditional plate‐based methods, including reduced laboratory time, fewer required dilutions to achieve readings within the dynamic range, enhanced ease in maintaining sterility during analysis, and suitability for lower Biosafety Level 1 (BSL‐1) conditions due to the contained nature of bacterial culturing within the Quanti‐Trays, rendering it a more accessible tool for widespread monitoring.

The IDEXX Colilert and Enterolert Quanti‐Tray methods were modified for assessing AMR based on previously validated protocols. Validation data showed that modified IDEXX methods correlate strongly with traditional plating (*r* = 0.95–0.967), rendering them an accessible alternative for utilities without advanced microbiological laboratories (Jimenez et al. 2025). Before sealing IDEXX Quanti‐Trays (IDEXX Laboratories, Westbrook, ME, USA; Cat. No. WQT‐2K), antibiotic stock solutions (440 μL of 32‐mg/mL vancomycin per 400‐mL secondary effluent) were spiked directly into IDEXX sample bottles to achieve the desired final concentrations. Prepared solutions in IDEXX bottles were transferred to the Quanti‐Tray and sealed using an IDEXX Quanti‐Tray Sealer PLUS (IDEXX Laboratories, Westbrook, ME, USA) and incubated horizontally for 24–28 h at 35°C ± 0.5°C ( *E. coli* ), or 41°C ± 0.5°C (*Enterococci*). Bacterial reactivation potential was assessed after extended incubation of 3–5 days. After incubation, trays were inspected under 365 nm ultraviolet (UV) illumination (6‐W Fluorescent UV Lamp, IDEXX Laboratories, Westbrook, ME, USA; Cat. No. WL160) to quantify fluorescent wells indicating bacterial growth. Colony‐Forming Units (CFU)/100 mL were determined using IDEXX Most Probable Number (IDEXX enumeration output) (MPN) Generator software (version 1.4.4; IDEXX Laboratories, Westbrook, ME, USA).

#### 2.4.1 Dilutions and Sample Preparation for IDEXX Analysis

Dilutions for bacterial enumeration were determined by examining data from previous research (WRF 5219) and preliminary results from this investigation so that counts remained within the dynamic range of the IDEXX system for accurate quantification (Table in SI). Prior to loading samples into Quanti‐Trays, wastewater samples were diluted using Milli‐Q purified water according to the schemes detailed in Table 3. Dilution schemes included 10× dilution (10‐mL wastewater, 90‐mL deionized water [DI] water) and 20× dilution (5‐mL wastewater, 95‐mL DI water). For all other samples, including those designed for AMR analysis, 100‐mL undiluted wastewater was used. Quenched subsamples were immediately analyzed to determine both total and resistant bacterial concentrations using the IDEXX Quanti‐Tray system.

**TABLE 3:** Posttreatment dilution scheme used for total enterococci (TE) enumeration to maintain IDEXX Enterolert counts within the instrument reporting range by disinfectant, dose, and contact‐time window.

Disinfectant	Concentration (mg/L)	Time (min)	Wastewater (mL)^a^	DI water
PFA	1	0–10	5–10	90–95
	2	0–2	5–10	90–95
PAA	2	0–30	5–10	90–95
	4	0–10	5–10	90–95
NaOCl (as Cl_2_)	2	0–30	5–10	90–95
	4	0–10	5–10	90–95

^a^ All other samples at contact times not listed in the table, in addition to VRE samples, were tested using undiluted (100%) full‐strength wastewater.

#### 2.4.2 Inactivation Assessment

Data were analyzed using an integrated concentration–time (ICT) approach, which has been shown to more accurately describe PFA inactivation under real wastewater conditions by capturing disinfectant demand and decay (Nyangaresi et al. 2026). The ICT values were computed using experimental data for each experimental condition to account for oxidant demand and decay. Instantaneous disinfectant concentration data were collected at discrete contact times and numerically integrated using the trapezoidal rule to obtain ICT in mg·min/L. This approach reflects the cumulative oxidant exposure experienced by microorganisms, incorporating the effect of temporal decay rather than assuming a constant concentration, as in conventional concentration × time (CT) models (Peleg 2021).

For NaOCl in ammonia‐rich, unnitrified secondary effluent, both FC and TC were measured using DPD methods. FC and TC were used as a QA/QC check (i.e., TC ≥ FC) and to confirm that residual was predominantly combined chlorine under ammonia‐rich conditions. For dose–response and ICT calculations, TC was used as the operational residual representing delivered oxidant exposure. We note that TC does not uniquely quantify monochloramine and may include other combined chlorine species (e.g., organic chloramines [OC]) in wastewater matrices.

PAA and PFA concentrations were quantified using the CHEMetrics Peracetic Acid SAM kit, employing the DPD‐KI colorimetric method. Given the inherent instability and rapid aqueous‐phase decomposition kinetics of peroxyacids, immediate analysis upon sample collection is required (Shengcen Zhang et al. 2020).

Microbial inactivation was expressed as log_10_ (*N*/*N*₀) (where *N*₀ and *N* are initial and surviving bacterial concentrations, respectively). *N*₀ was determined using batch‐specific time‐zero counts. Because microbial quantification estimates exhibit stochastic error, especially at low counts and across dilutions, uncertainty in *N*₀ propagates directly to LR and slope (S) estimates (Jimenez et al. 2025; Sercu et al. 2010). Early‐time deviations (e.g., apparent “shouldering”) were interpreted conservatively as the combined effects of series‐event inactivation and measurement uncertainty in *N*₀ and *N*; accordingly, model fitting was restricted to empirically linear ICT ranges to minimize bias (Peleg 2021).

Log reductions were plotted against corresponding ICT values to establish dose–response relationships. Regression models with segmentation accounting for biphasic inactivation were used to assess the linearity and slope of inactivation trends, providing a basis for comparing oxidative efficacy and stability profiles. This approach allows a kinetic comparison and identification of operational ICT thresholds associated with robust inactivation performance for target organisms.

#### 2.4.3 Relative Resistance and Repair/Reactivation Potential Assessment

To determine whether AMR influenced disinfection sensitivity, “Relative Resistance” was assessed by performing linear regression of the Log_10_ reduction of VRE (*y*‐axis) against the Log_10_ reduction of TE (*x*‐axis) for samples across all tested doses and times. The resulting slope was compared with a theoretical parity slope of 1.0. Although a slope of 1.0 indicates conserved sensitivity across the disinfectant range, a slope significantly less than 1.0 (S < 1.0) would indicate that VRE is more resistant than the total TE population, and a slope significantly greater than 1.0 (S > 1.0) would indicate that VRE is less resistant than the total TE population.

“Repair Potential” was evaluated by comparing Log_10_ reductions obtained with standard incubation (24 h) against those obtained via extended incubation (3–5 days). Linear regression was applied to the Log_10_ reduction of standard incubation (*x*‐axis) versus extended incubation (*y*‐axis). A segmented approach was employed as needed to evaluate if repair potential varied between low‐ICT and high‐ICT regimes.

For both the relative resistance and repair potential, the standard error of the slope (SE), coefficient of determination (*R* ^2^), and *p*‐values were calculated. Hypothesis testing was performed using a two‐tailed *t*‐test (shown in Equation 3). Specifically, the null hypothesis (*H*_0_) assumed a slope of 1.0 (no significant difference between bacterial AMR or incubation protocols).

The *t*‐statistic was calculated as follows:

(3) $$t=\frac{\left(S_{\mathrm{obs}}-H_{0}\right)}{\mathrm{SE}}$$

where

- *t* = *t*‐statistic (dimensionless)
- $S_{\mathrm{obs}}$ = observed regression slope (dimensionless, ratio of log_10_ reductions)
- $H_{0}$ = hypothesized slope (dimensionless; here, $H_{0}$ = 1.0)
- SE = standard error of the slope (dimensionless)

Additionally, the reactivation potential (ΔΔlog) framework derived from paired log_10_ reduction measurements obtained under standard and extended incubation conditions was developed (Equation 4). For each experimental batch, primary inactivation was expressed as a log_10_ reduction:

(4) Δlog=LR

where

- Δlog = log_10_ reduction (dimensionless)
- LR = log_10_ reduction in microbial concentration (dimensionless)

Postincubation assessments were conducted following reactivation or regrowth phases under nutrient‐limited conditions (Equation 5).

Reactivation was expressed as a change in log reduction over time:

(5) $$\text{ΔΔlog}={\text{Δlog}}_{\text{Extended}}-{\text{Δlog}}_{\text{Standard}}$$

where

- ΔΔlog = reactivation index (RI) (dimensionless)
- Δlog_Extended_ = log reduction after extended incubation (dimensionless)
- Δlog_Standard_ = log reduction after standard incubation (dimensionless)

This paired‐difference approach was used to minimize bias associated with the loss of selective inhibition in Enterolert after prolonged incubation (> 27 h). This approach also mathematically removes variability arising from disinfectant decay, dose delivery, and water quality, thereby isolating postdisinfection microbial recovery from initial inactivation. Because the extended and standard incubation measurements originated from the same sample and were processed in identical media, systematic artifacts, such as increases in background fluorescence, partial inhibitor breakdown, or nontarget recovery, are assumed to affect both incubation periods similarly. Subtracting the standard value therefore removed shared measurement drift and isolated the net increase in viable cells attributable to biological reactivation rather than methodological instability.

Building upon the reactivation potential framework, reactivation profiles derived from ΔΔlog values enable comparative evaluation of disinfectant performance by distinguishing reversible injury or VBNC states from more durable inactivation outcomes (Ding et al. 2023; Yin et al. 2023). Specifically, two key patterns emerge in these profiles:

- Operational durability threshold: Profiles exhibiting a maximum reactivation level followed by a decline at elevated ICT values indicate an exposure–response region where recoverability decreases. This inflection point is interpreted operationally as a transition toward more robust (durable) inactivation, consistent with reduced recovery at higher exposures (Ding et al. 2023; Huang et al. 2013).
- Persistent reactivation: Profiles showing continued increase in reactivation with ICT indicate suboptimal performance, as exposure conditions are insufficient to suppress recoverability, consistent with persistent sublethal injury or VBNC‐associated states detectable via extended incubation (Fan et al. 2024; Rocher et al. 2021; Yin et al. 2023).

This structured evaluation provides a consistent and objective framework to compare disinfectants and identify exposure conditions associated with robust inactivation performance in wastewater treatment applications (Ding et al. 2023).

### 2.5 Data Analysis: Statistical and Analytical Methods

#### 2.5.1 Data Analysis Environment

Data processing, statistical analysis, and visualization were performed using Python (version 3.10.16; Python Software Foundation, Wilmington, DE, USA). Data cleaning, transformation, and management were conducted using the pandas (version 2.0.0) and NumPy (version 1.26.3); SciPy (version 1.7) was used for regression and *t*‐tests, and Matplotlib (version 3.4) for plotting. The statsmodels library (version 0.13+) was utilized for correlation analyses. Statistical significance was defined as *p* < 0.05.

#### 2.5.2 Inclusion/Exclusion Criteria

Only data points with bacterial counts above the limit of detection (LOD) (CFU > 1) were included. Observations in the tailing regions of inactivation curves were retained to assess the persistence and repair potential of survivors.

The complex interaction models for disinfectant kinetics were based on the statistical methodology originally implemented in Tableau, which allowed dynamic filtering, outlier inspection, and correlation tracking between log‐reduction trends and batch variability, facilitating transparent interpretation of the statistical results. These dashboards also facilitated direct visual comparison of disinfection slopes, batch effects, and nonlinear behavior outside the linear inactivation range.

#### 2.5.3 Statistical Analysis of Inactivation and Reactivation Kinetics

Slope‐based kinetics to delta‐based performance metrics using ordinary least squares (OLS) and analysis of covariance (ANCOVA) were used to characterize the primary inactivation rates by identifying high‐resolution interactions between ICT, dose, incubation type (standard/extended), and bacteria type (TE/VRE). These models exhibited high explanatory power (*R* ^2^ ~ 0.95), capturing batch‐specific nuances like the “Lower Dose Advantage” observed for chloramines.

#### 2.5.4 Range Selection for Linear Response Analysis

Dose–response analyses were restricted to empirically validated linear ICT ranges corresponding to clearly distinguishable log‐reduction behavior:

- PAA: ICT > 20 mg·min/L
- PFA: ICT < 28 mg·min/L
- TC: ICT < 90 mg·min/L

Model fitting occurred within the monotonic, near‐linear response regime, avoiding saturation or tailing effects that could obscure disinfectant efficacy comparisons.

## 3 Results

Table 4 presents characteristics of three GLWA WRRF secondary effluent samples used in batch experiments. Microbial analysis results were generally above the detection limit for the methods used, except for a limited number of instances (Table 5).

**TABLE 4:** Wastewater quality parameters for the secondary‐effluent batches used in disinfection experiments.

Parameter	Batch		
	A	B	C
Date collected	4/13/25	5/3/25	5/4/25
TSS (mg/L)	3.5	5.6	13.0
COD (mg/L)	31	52	49
pH	7.6	7.2	7.47
Temperature (°C)	19.8	21.9	21.7

**TABLE 5:** Conditions under which CFU measurements were below the detection limit (CFU < 1 per 100 mL). All samples were undiluted.

Disinfectant	ICT (mg·min L^−1^)	Initial target dose (mg L^−1^)	Contact time (min)	Residual (mg L^−1^)	Incubation	Bacteria	Sample	Notes
PAA	99.15	4	30	3.05	Standard	VRE	A	Nondetect at high ICT
PAA	99.15	4	30	3.05	Extended	VRE	A	Nondetect repeated across incubation types
PFA	7.29	1	10	0.58	Standard	VRE	B	Nondetect at low ICT
PFA	8.04	2	5	1.43	Standard	VRE	B	Nondetect across low–moderate ICT
PFA	7.29	1	10	0.58	Extended	VRE	B	Consistent nondetect across incubation types
NaOCl	6.93	2	30	0.17	Standard	VRE	A	Nondetect at low residual chlorine

### 3.1 Disinfectant‐Specific Inactivation Kinetics, Relative Resistance, and Reactivation Profiles

#### 3.1.1 PFA

**FIGURE 2:**
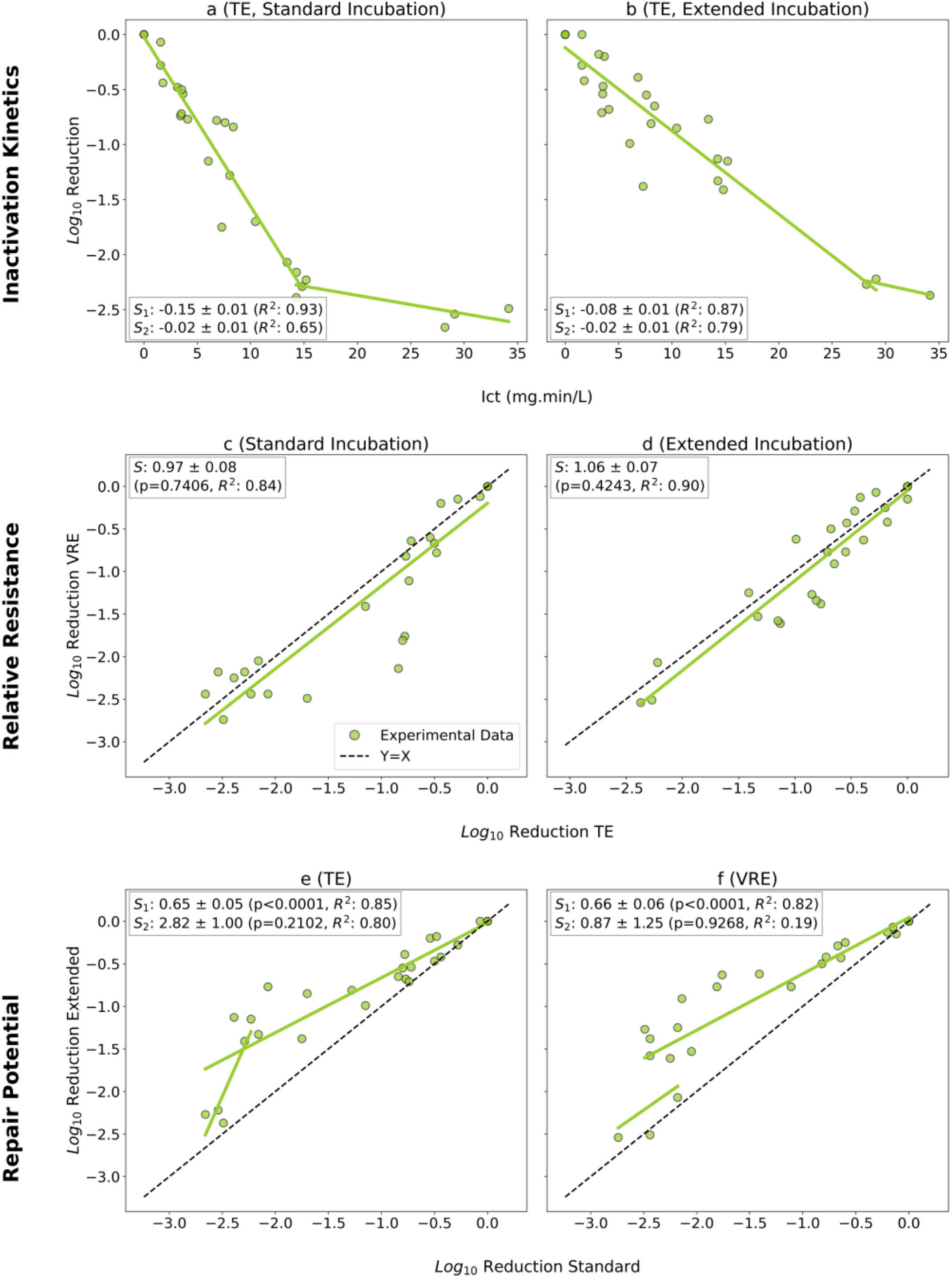
PFA disinfection performance for TE and VRE. (a–b) Inactivation kinetics: TE log_10_ reduction versus ICT under (a) standard and (b) extended incubation. (c–d) Relative resistance: VRE versus TE log_10_ reductions. Data convergence on the line of equality (*y* = *x*) indicates PFA is nonselective for resistance (*p* > 0.05). (e–f) Recoverability/reactivation: extended versus standard incubation comparisons for TE (e) and VRE (f). ICT, integrated concentration–time; PFA, performic acid; TE, total enterococci; VRE, vancomycin‐resistant enterococci.

Statistical analyses presented in Section S5 (Table S2) indicated that the relationship between log reduction and ICT for PFA was not significantly different (*p* > 0.26) for the initial PFA target dose or for the three samples tested for any of the four microbial analyses conducted. Consequently, data were pooled (Figure 2).

PFA exhibited consistent, rapid, linear inactivation kinetics for all microbial analyses, followed by tailing at higher ICT; for TE, tailing was delayed under extended incubation, and VRE remained proportional to TE (Figure 2). Figure 2a,b illustrates a steep, consistent Log_10_ reduction for TE with increasing ICT under standard and extended incubation conditions, followed by a reduced rate at elevated ICT (> 28 mg·min/L). For TE under standard incubation, the initial inactivation slope was −0.15 ± 0.01 L/mg·min with an *R* ^2^ of 0.93. Under extended incubation, the initial inactivation slope was −0.08 ± 0.01 L/mg·min with an *R* ^2^ of 0.87. Extended incubation shifted the rapid‐to‐tailing transition from ~15 to 30 mg·min/L for TE. An apparent short initial lag was observed at ICT < 2 mg·min/L. Figure 2c,d compares the Log_10_ reduction for VRE with the Log_10_ reduction for TE for standard and extended incubation. The dotted line represents a one‐to‐one relationship, where VRE and TE decline at the same rate, whereas the solid line illustrates the statistical relationship. Results show that VRE and TE Log_10_ reduction are not statistically different and vary proportionally, indicating that VRE is inactivated at the same rate as TE, whether measured at standard or extended incubation. Under standard incubation (Figure 2c), the slope comparing Log_10_ reduction for VRE to Log_10_ reduction for TE was 0.97 ± 0.08, which was not significantly different from 1. Similarly, under extended incubation (Figure 2d), the slope was 1.06 ± 0.05 L/mg·min, also not significantly different from 1, indicating similar susceptibility between resistant and total bacteria.

Figure 2e,f assesses reactivation by plotting Log_10_ reduction for extended incubation versus Log_10_ reduction for standard incubation for TE and VRE, respectively. Results indicate reactivation (lower Log_10_ reduction for extended than for standard incubation) over most of the incubation range. These reductions were similar at the highest levels of standard incubation Log_10_ reduction, indicating reactivation could be avoided when higher levels of inactivation, reflected by standard incubation, are achieved. The relationship for extended versus standard incubation is similar for TE and VRE, indicating TE and VRE reactivation occurred at the same rate. Reactivation was negligible at an applied ICT > 30 mg·min/L PFA.

#### 3.1.2 PAA

**FIGURE 3:**
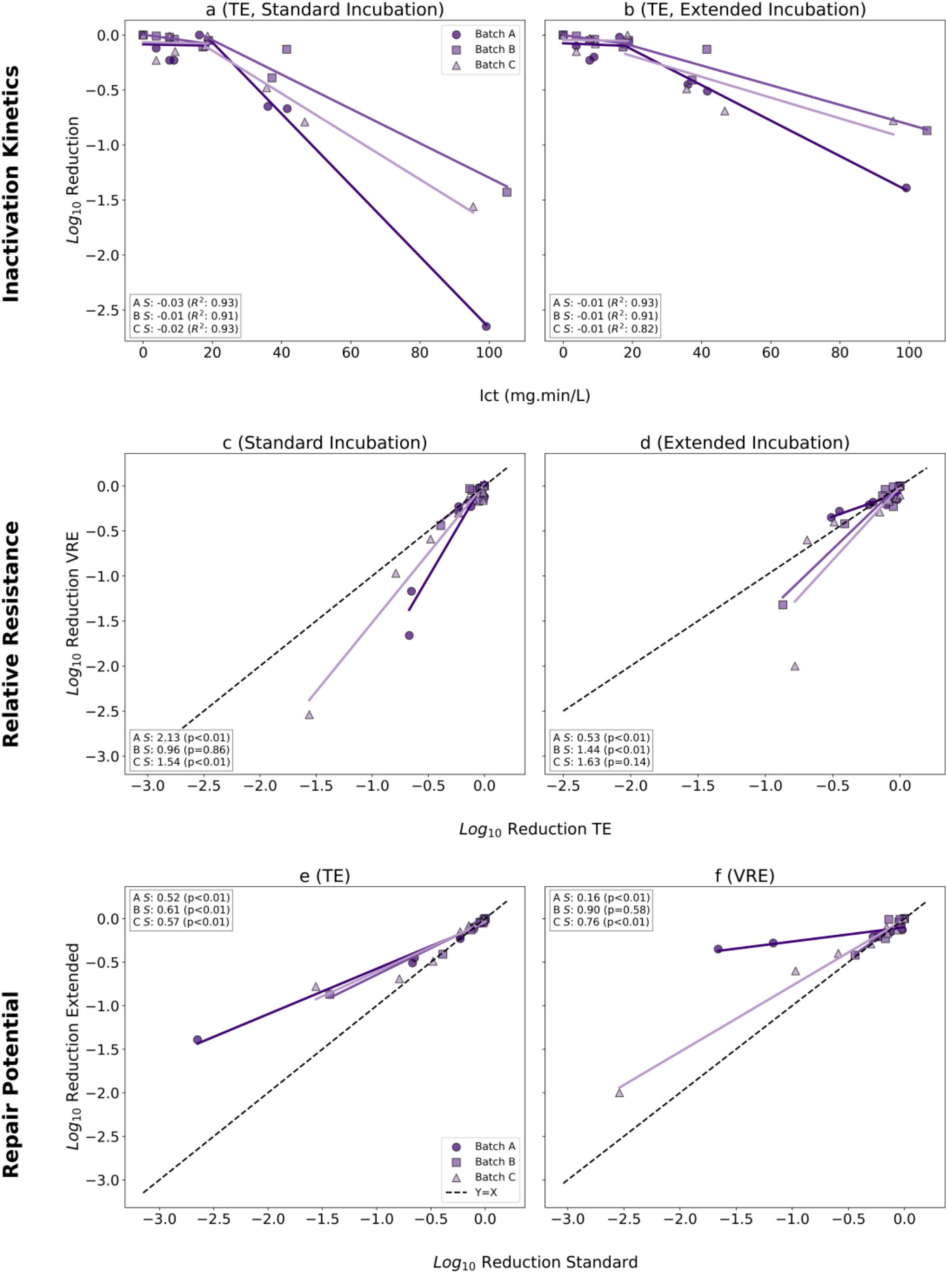
PAA disinfection performance for TE and VRE. (a–b) Inactivation kinetics: TE log_10_ reduction versus ICT under (a) standard and (b) extended incubation, showing shouldering at low ICT. (c–d) Relative resistance: VRE versus TE log_10_ reductions. (e–f) Recoverability/reactivation: extended versus standard incubation comparisons for TE (e) and VRE (f). ICT, integrated concentration–time; PAA, peracetic acid; TE, total enterococci; VRE, vancomycin‐resistant enterococci.

Statistical analysis of PAA data indicated that the Log_10_ reduction at matching ICTs for initial target doses of PFA of 2 and 4 mg/L was not significantly different (*p* = 0.85) across all experimental batches and incubation types, as expected. Thus, results were pooled according to ICT. The results were statistically different, however, among the batches (*p* = 0.008), which are plotted separately in Figure 3.

PAA demonstrated markedly slower and more batch‐variable inactivation kinetics than PFA, consistently requiring higher ICT to secure equivalent reductions (Figure 3a,b). A distinctive shouldering pattern was observed at low ICT (< 20 mg·min/L), with minimal initial reductions before steeper declines at higher ICT.

The relative resistance profile for PAA showed greater variability than for PFA and indicated batch‐dependent differences between VRE and TE (Figure 3c,d). Overall, VRE log_10_ reductions were generally comparable with, and in some cases greater than, TE log_10_ reductions across the interpretable range. For Batch A, high‐ICT VRE endpoints were limited in the relative‐resistance panels.

The repair potential analysis for PAA (Figure 3e,f) showed a pronounced separation between standard and extended incubation data. For TE, extended‐versus‐standard slopes were < 0.61 (*p* < 0.001), indicating substantial posttreatment recoverability; VRE showed a similar pattern, with the magnitude varying by batch.

#### 3.1.3 NaOCl

**FIGURE 4:**
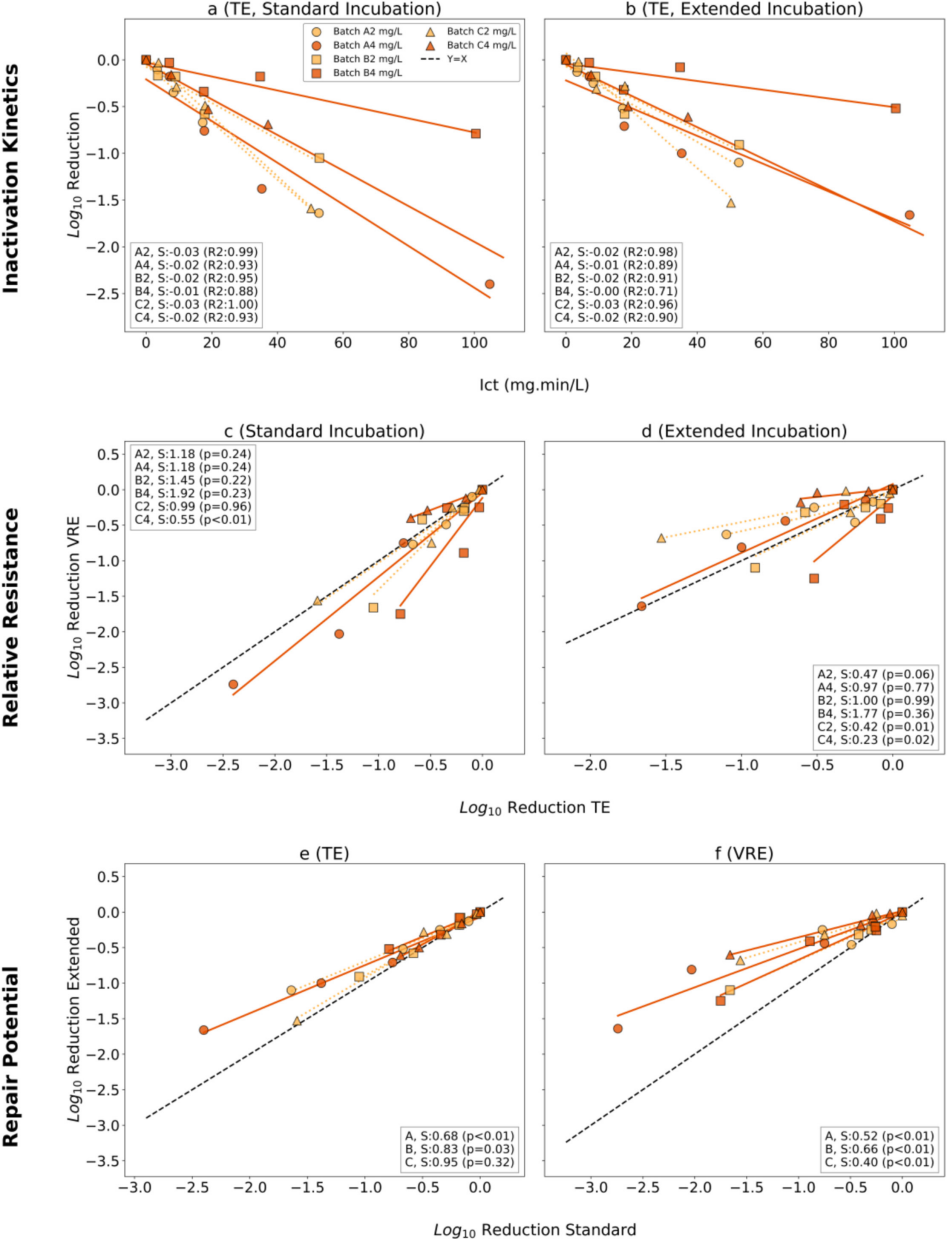
NaOCl disinfection performance for TE and VRE using total chlorine (TC)–based ICT. (a–b) Inactivation kinetics: TE log_10_ reduction versus ICT by dose. (c–d) Relative resistance: VRE versus TE log_10_ reductions. (e–f) Recoverability/reactivation: extended versus standard incubation comparisons for TE and VRE. ICT, integrated concentration–time; NaOCl, sodium hypochlorite; TC, total chlorine; TE, total enterococci; VRE, vancomycin‐resistant enterococci.

As noted, NaOCl performance was evaluated using TC data (ICT ≤ 100 mg·min/L) (Figure 4), rather than FC (ICT ≤ 12 mg·min/L), because NaOCl in the presence of ammonia forms chloramines with a formation rate constant on the order of 10^6^ M^−1^ s^−1^, ensuring near instantaneous conversion of NaOCl to monochloramine in wastewater matrices containing elevated ammonia (Qiang and Adams 2004). This is important because there is an analytical artifact known as DPD monochloramine breakthrough, in which monochloramine produces a false positive FC signal (Gehr et al. 2009). The agreement between FC and TC trends confirms that Method 10245 is impacted by this effect; comparison of FC (Figures S3a and S3b) and TC (Figures S3c and S3d) decay and inactivation kinetics indicated that applied chlorine doses of 2 and 4 mg/L resulted in the immediate formation of combined chlorine residuals. The “Free” signal captured only ~15%–25% of the actual oxidant pool, artificially steepening inactivation slopes by a factor of 10 (Gehr et al. 2009). Given the ammonia concentration (~17 mg/L), the applied chlorine doses (2–4 mg/L) result in chlorine‐to‐nitrogen weight ratios (0.12:1 and 0.24:1) far below the threshold for breakpoint chlorination, indicating instantaneous conversion of FC to monochloramine. Figures S8–S11 comparing the initial apparent FC residual (~0.45 mg/L) with the corresponding TC (~1.85 mg/L) indicate a breakthrough magnitude of ~24%, confirming that the FC signal is a false positive. Because the applied chlorine doses are stoichiometrically overwhelmed by ammonia, FC are unlikely to coexist, and monochloramine is the predominant disinfecting species. TC decay curves track the applied doses and display a monotonic decline representative of oxidant availability.

Of the three disinfectants evaluated, NaOCl exhibited the least consistent inactivation kinetics, characterized by pronounced scatter and noticeable differences in outcomes driven by initial dose (*p* < 0.05), experimental batch (*p* < 0.001), and incubation duration. Although bacteria type was not significant in the aggregate model (*p* = 0.98), a more detailed subanalysis revealed that it was a significant factor in Replicates B (*p* = 0.018) and C (*p* = 0.048), particularly under extended incubation conditions. In these runs, VRE exhibited a different inactivation trajectory compared with TE, a finding masked in the global model by the lack of effect in Replicate A. This replicate‐dependent sensitivity of the bacteria type effect, where AMR may confer a survival advantage under specific water quality or physiological conditions, further justifies the decision to present Cl‐Total data without pooling, stratifying by dose, replicate, and incubation type to preserve these localized findings.

Inactivation kinetics for TE and VRE followed a pseudo‐first‐order Chick–Watson relationship when plotted against ICT that reached 100 mg·min/L (*R* ^2^ = 0.88–1) (Figure 4a,b). Inactivation rate constants (*k*) for TE ranged from 0.01 to 0.03 L/mg·min. A critical examination of the inactivation curves indicated that NaOCl treatments featured slight initial shouldering alongside sharply reduced inactivation between 5 and 10 min (Figure 4a,b). A significant dose effect was observed at identical ICT values: The 2‐mg/L dose consistently achieved higher log reductions (slopes of −0.02 to −0.03) than the 4‐mg/L dose (slopes of −0.01 to −0.02).

The relative resistance analysis for NaOCl (Figure 4c,d) demonstrated interesting trends. Batches that displayed larger inactivation rate constants, C2 and C4 (Figure 4a,b), indicated significant differences between TE and VRE, with VRE often showing lower log reduction or more reactivation than TE for comparable ICTs. Conversely, similar TE‐VRE inactivation was observed in batches demonstrating slow or no inactivation: A4 and B4 with slopes of −0.01 and 0.00 (Figure 4a,b).

Inspection of reactivation data indicated that log reduction of extended incubation versus standard incubation was dose‐independent. Therefore, reactivation data were pooled across doses. NaOCl displayed the most rapid and significant reactivation potential, being initiated at low ICT and increasing with ICT (Figure 4e,f). The most notable feature was the pronounced separation between standard and extended incubation, particularly for VRE, indicating substantial microbial reactivation and potential for regrowth, with multiple extended incubation points showing near‐zero log reduction.

## 4 Discussion

### 4.1 Disinfectant Performance

By integrating ICT‐based kinetics, relative resistance assessments, and reactivation analyses under standard and extended incubation, these findings highlight substantial differences in the reliability of disinfection delivered by each oxidant at the tested doses. Results of this study indicate advantages for PFA disinfection relative to PAA and NaOCl within the scope of this study.

PFA consistently demonstrated superior disinfection performance, characterized by rapid, robust inactivation. The steep, linear inactivation kinetics observed at ICT < 28 mg·min/L (Figure 2a,b) align with previous findings that highlight PFA efficiency and sustained microbial inactivation (Maffettone et al. 2020) (Table S2).

VRE were inactivated in proportion to TE, whether evaluated by standard or extended incubation, as evidenced by Log_10_ reduction ratios not statistically different from 1 (Figure 2c,d).

The insensitivity of PFA kinetics to its initial concentration and AMR, coupled with its robustness against measured wastewater quality parameters (Table S2 and Figure S6), underscores its reliability across varying operational and environmental conditions (Bagagnan et al. 2024).

The requirement for an ICT‐based kinetic framework for PFA in this study was fundamentally driven by the rapid decomposition of PFA in secondary effluent matrices, consistent with the known instability and fast aqueous‐phase decay of peroxyacids (Shengcen Zhang et al. 2020). Although this instability necessitates on‐site generation of PFA (Ding et al. 2023; Gehr et al. 2009), it serves as a critical environmental safeguard, unlike the persistent chloramine residuals observed in NaOCl trials, PFA residuals decay within minutes into benign byproducts such as water, oxygen, and dilute FA (Gehr et al. 2009). This transient nature of PFA minimizes the risk of downstream ecological toxicity in receiving water bodies, where even low concentrations of residual chlorine are known to be acutely lethal to aquatic invertebrates and fish (Blatchley 2023).

In contrast, PAA exhibited a more variable and complex disinfection profile than PFA, likely because its effective exposure is more sensitive to wastewater matrix effects, particularly suspended‐solids/floc interactions that increase oxidant demand, accelerate PAA decay, and can contribute to particle‐associated protection/aggregation. In our batches, variability coincided with differences in TSS (3.5–13 mg/L) and showed a moderate but not statistically significant negative association for VRE under extended incubation (Figure S6). The influence of suspended solids on PAA decay and disinfection performance is documented (Amerian et al. 2019; Domínguez Henao et al. 2018). However, strong causal relationships between wastewater matrix characteristics and disinfectant performance could not be established here because wastewater characterization was limited and the number of independent samples was small.

The slower inactivation kinetics and the observed shouldering pattern of PAA at lower ICTs (Figure 3a,b) suggest a phenomenological kinetic feature, consistent with multistep disinfection kinetics and/or matrix effects. Operationally, it indicates that higher ICTs were needed to achieve robust reductions within the tested conditions. This shouldering effect could also be attributed to the influence of suspended solids on PAA decay kinetics or the initial degradation of microbial flocs, which can release entrapped bacteria and lead to varied observed net inactivation rates. Results also suggest that VRE inactivation under PAA was generally comparable to, and in some cases greater than, TE inactivation across the interpretable range (Figure 3c,d). This pattern suggests no consistent enrichment of culturable VRE relative to TE under the tested PAA conditions. Furthermore, the observed increased inactivation of VRE relative to TE represents an advantage for PAA, if confirmed, suggesting the need for further experimentation if PAA disinfection were pursued.

Because high‐ICT VRE endpoints for Batch A were limited in the relative‐resistance comparison, interpretation of VRE behavior at the upper exposure range should be made cautiously.

The secondary effluent pH values observed across batches (7.2–7.6; Table 4) place all disinfectants in a regime where modest pH differences can influence aqueous form, decay behavior, and therefore the effective delivered exposure (Nyangaresi et al. 2026). This is particularly important for chlorine‐based disinfection in ammonia‐rich effluents, where FC is rapidly converted, and the operative disinfecting residual is primarily combined chlorine, consistent with our decision to use TC‐based ICT for cross‐oxidant comparisons in this ammonia‐rich matrix.

Differences in disinfection performance between PAA and PFA are driven by oxidant speciation (pH‐dependent acid–base form), intrinsic reactivity/selectivity, and wastewater matrix interactions (e.g., oxidant demand and particle‐associated protection) (Luukkοnen and Pehkonen 2016). PAA may require a period of measurable exposure and a specific threshold level of cellular damage before rapid culturability loss occurs, resulting in delayed inactivation. This is attributed to the combined effects of peracid activity (including selective oxygen‐transfer reactivity toward reduced‐sulfur targets) and matrix‐dependent consumption/shielding, rather than molecular weight alone (Wang et al. 2023). The disinfection mechanisms of PAA involve oxidative reactions that can include intracellular oxidative stress after membrane penetration, as reported for peroxyacids in comparative mechanistic studies, and sublethal injury, potentially leading to a VBNC state rather than immediate cell death (Truchado et al. 2021).

PFA and PAA addition for disinfection also results in the addition of oxygen‐demanding constituents to the effluent. The theoretical oxygen‐demand (ThOD) contribution associated with peracid disinfectants is a function of the entire equilibrium mixture, rather than the active peracid alone (Table 6). Although the stoichiometric oxygen demand of PAA is 0.63 mg O_2_/mg, the total effluent impact is dominated by the presence of the parent AA, which possesses a higher COD factor of 1.07 mg O_2_/mg. Because dosed commercial PAA solutions are equilibrium mixtures of PAA, acetic acid, and hydrogen peroxide, formulation composition influences the net oxygen‐demanding load. Conversely, PFA exhibits a unique internal oxygen balance (CH_2_O_3_ → CO_2_ + H_2_O), rendering the molecule stoichiometrically COD‐neutral. However, the accompanying FA in the PFA equilibrium solution contributes a residual COD of 0.35 mg O_2_/mg, so practical dosing of PFA stock and generator output introduces oxygen demand via FA and potentially H_2_O_2_, depending on formulation. Consequently, on a per mg active peracid delivered basis, the net oxygen‐demand contribution associated with PAA‐based dosing is estimated to be ~1.2–5.3× higher than PFA‐based dosing when expressed as apparent dichromate COD (including H_2_O_2_ interference), assuming PFA generator output contains ~10‐ to 16‐wt% active PFA and residual FA/H_2_O_2_ carryover is bounded using a stoichiometric equilibrium framework (Section S6 and Table S5). On a ThOD‐only basis (excluding peroxide interference), the corresponding ratio is higher (~1.7–10.2×; Supporting Information). These results highlight the necessity of accounting for the entire chemical matrix when assessing the environmental and regulatory footprint of peracid‐based disinfection. Accounting for COD increase is important for facilities operating near stringent discharge limits because residual organic acids (acetic or formic) can remain in the solution after disinfection and contribute to oxygen demand until they biodegrade. Because residual peracids or H_2_O_2_ can bias dichromate COD measurements, interpretation of measured COD should account for potential oxidant interference.

**TABLE 6:** Stoichiometric oxidation reactions and theoretical COD factors for the individual constituents of peracid equilibrium solutions. All COD factors are expressed as mg O_2_ per milligram of the specific component.

Component	Stoichiometric oxidation reaction	COD factor (mg O_2_/mg)
AA	CH_3_COOH + 2O_2_ → 2CO_2_ + 2H_2_O	1.07
PAA	CH_3_COOOH + 1.5O_2_ → 2CO_2_ + 2H_2_O	0.63
FA	HCOOH + 0.5O_2_ → CO_2_ + H_2_O	0.35
PFA	HCOOOH + 0O_2_ → CO_2_ + H_2_O	0.00
Hydrogen peroxide (H_2_O_2_)^a^	H_2_O_2_ → H_2_O + 0.5O_2_ (interference)	0.47

^a^ H_2_O_2_ factor reflects apparent COD contribution via dichromate analytical interference rather than carbonaceous oxygen demand.

Experimental results indicate that PAA treatment can increase effluent COD (increases of 15% to ~28 mg O_2_/L at 5‐ to 10‐mg/L doses over 5–20 min) (Table 7). PAA disinfection elevates effluent COD, stemming from organic oxidation and AA byproducts, potentially impacting downstream treatment or reuse (Cavallini et al. 2013). Low PAA doses (2–5 mg/L) may trigger substantial regrowth (> 50‐fold vs. untreated), fueled by byproduct nutrients (AA and bioavailable carbon), imposing selection pressure on antibiotic‐resistant strains. This increases the risks of biofilm formation and posttreatment bacterial blooms (Bagagnan et al. 2024). In contrast, PFA introduces the least amount of organic carbon among the common peracids on a molar basis (Luukkοnen and Pehkonen 2016). PFA decomposes primarily into FA, water, and oxygen or undergoes radical degradation into carbon dioxide and water. FA residuals at standard disinfection dosages are considered environmentally benign, nontoxic to aquatic life, and far less problematic regarding organic loading than AA (Bagagnan et al. 2024).

**TABLE 7:** Literature‐reported increases in COD following peracetic acid (PAA) treatment. COD was measured as the bulk organic response to PAA‐induced oxidation and the release of partially oxidized or bioavailable organic matter. Initial wastewater characteristics, including microbial and organic matter concentrations, varied across experiments and contributed to uncertainty when comparing COD outcomes.

PAA dose	Contact time	Reported COD increase	Mechanistic interpretation	Wastewater type	Study/citation
10 mg/L	20 min	15% average increase	Oxidation of recalcitrant extracellular organic matter, generating smaller and more bioavailable organic fragments	Wastewater effluent	(Cavallini et al. 2013)
10 mg/L	10 min	27.6 mg O_2_/L increase	PAA oxidation with measurable contribution from AA released during PAA decomposition	Wastewater effluent	(Cavallini et al. 2013)
5 mg/L	5–10 min	Statistically significant COD increase	Organic modification and release of oxidized organics from suspended/dissolved solids due to PAA reactions	Simulated combined sewer overflow	(Eramo et al. 2017)

Chloramines generated from NaOCl consistently demonstrated the most complex disinfection profile, characterized by highly variable and generally slower inactivation kinetics (Figure 4a,b). Experimental data for various batches and doses were not pooled due to statistically significant variation in inactivation efficiency (*p* < 0.05). The batch effect (notably the extreme resistance of Batch B) indicates the secondary effluent matrix, likely due to variations in organic matter reflected by the largest measured COD (52 mg/L) or the increased suspended solids TSS (5.6 mg/L), alters apparent chloramination performance. Further, the dose effect indicated that an initial NaOCl dose of 2 mg/L (as Cl_2_) provided greater inactivation than 4 mg/L at the same ICT; pooling these disparate data series would mask these differences, leading to inaccurate disinfection assessments. The counterintuitive observation that lower initial doses sometimes achieved similar or greater inactivation at equivalent ICTs may reflect differences in oxidant speciation and effective disinfectant fraction across dose regimes. Because ICT was calculated from TC, the formation of less biocidal combined‐chlorine species could inflate ICT without proportional germicidal efficacy. This pattern may be driven by rapid consumption of higher initial doses by matrix constituents, conversion to less potent combined‐chlorine species, and/or differences in the reversibility of damage under high initial oxidative shocks. In high‐ammonia environments, a 2‐mg/L chlorine dose is expected to favor predominantly monochloramine formation, whereas a 4‐mg/L dose can generate localized hypochlorous acid (HOCl) during initial mixing, promoting the rapid formation of OC from dissolved organic nitrogen (Y. Zhang et al. 2021). These OCs, indistinguishable from inorganic chloramines by standard total chlorine residual assays, exhibit negligible biocidal activity, thereby inflating apparent ICT values without commensurate germicidal efficacy (Y. Zhang et al. 2021). Direct quantification of monochloramine versus OC was not performed; therefore, this interpretation remains a mechanistic hypothesis consistent with the observed TC‐based ICT behavior.

Higher initial oxidative concentrations exacerbate inactivation tailing through multiple mechanisms, including stress‐induced resistant subpopulations and penetration‐limiting surface reactions. The 4‐mg/L dose can oxidize and harden floc surfaces, forming diffusive barriers that hinder monochloramine penetration (Behnke et al. 2011). Biologically, such concentrations, unlike the more gradual metabolic disruption from 2‐mg/L dosing, induce oxidative shock and sublethal injury, promoting stress‐response states and delayed inactivation consistent with VBNC‐like behavior. Mathematically, such patterns align with the Chick‐Watson model, where the coefficient *n* < 1 for chloramines underscores the dominance of contact time over concentration in VRE inactivation (Hassen et al. 2000).

These inherent inefficiencies of higher initial monochloramine concentration can be mitigated through various engineering and dosing strategies. High‐intensity mixing (e.g., *G*ˉ ≈ 10,000 s^−1^) disperses localized demand hotspots, improving disinfectant distribution (Y. Zhang et al. 2021). Similarly, hydrodynamic optimization, specifically turbulence‐driven mixing that reduces short circuiting and improves contact uniformity, further enhances overall CT efficiency (Fallah et al. 2025). Sequential “step dosing” strategies, where disinfectant is added incrementally at multiple points, can improve disinfection performance by preventing localized high concentration zones that otherwise promote unproductive speciation, rapid oxidant consumption, and surface passivation effects. By smoothing the concentration profile, step dosing increases effective penetration into flocs and reduces the formation of resistant subpopulations associated with early oxidative shock (Behnke et al. 2011). Under such optimized dosing regimes, the inverse dose–response disparity commonly seen with monochloramine, where higher initial concentrations yield lower log reductions at equivalent ICT, is diminished because inactivation becomes controlled by sustained exposure rather than inhibited by high dose surface reactions or sublethal injury (Wahman et al. 2009).

Hydraulic short‐circuiting amplifies the drawbacks of high‐dose, short‐contact disinfection strategies, making it essential to design contactors that approximate plug‐flow behavior. Use of multiple stages, typically three or more, and high length‐to‐diameter aspect ratios reduces dispersion and ensures that all fluid elements achieve the intended disinfectant contact time.

Monochloramine is strongly temperature‐ and pH‐dependent. Optimal formation occurs at pH 7–8 with chlorine‐to‐ammonia ratios ≤ 5:1; avoiding alkaline conditions that accelerate decomposition pathways is critical for preserving residual potency. These dependencies reflect the broader principle that disinfectant stability and microbial inactivation kinetics must be evaluated under dynamically changing chemical conditions (Peleg 2021).

The apparent slight shouldering effects observed are common in NaOCl disinfection and can be attributed to factors such as bacterial clumping, the presence of resistant subpopulations, shielding by particles, and the inherent decay of the disinfectant in complex matrices. The relative resistance analysis of NaOCl data revealed batch‐to‐batch variability in VRE inactivation, underscoring its inconsistent performance. In batches exhibiting higher inactivation rate constants (Figure 4a), VRE predominantly demonstrated significantly greater resistance relative to TE (*p* < 0.05), evidenced by slower inactivation kinetics (S < 0.5 in Figure 4d) or elevated reactivation (S < 0.6 in Figure 4f) compared with TE, potentially favoring selective enrichment of vancomycin‐resistant strains (M. Wang et al. 2022).

The most critical drawback of NaOCl was its substantial and sustained VRE reactivation potential. The repair potential graphs for NaOCl showed the most pronounced separation between standard and extended incubation data for VRE, with many extended incubation points approaching zero Log_10_ reduction. The log reduction ratio analysis, revealing VRE to be less susceptible to inactivation or significantly more prone to reactivation than TE (S < 1, *p* < 0.001 in Figure 4f), is consistent with documented limitations of conventional chlorination against resistant strains and its tendency to induce VBNC states and regrowth in *E. coli* after chlorine disinfection (M. Wang et al. 2022). The presence of VRE in sewage treatment plants employing chlorination further highlights this concern.

### 4.2 VBNC Formation and Reactivation: Implications for AMR Management

Across all disinfectants, extended incubation revealed meaningful levels of sublethal injury and microbial reactivation (Figures 2, 3, 4, Rows e–f). PFA exhibited the least reactivation, and only below a short ICT range. PAA and NaOCl showed persistent and dose‐dependent reactivation throughout the tested ICT ranges. Reactivation occurred with lower overall rates of inactivation by PFA, corresponding to lower ICT, but reactivation was negligible at ICT above 28–34 mg·min/L (Figure 2e,f). This pattern is consistent with predominantly nonrecoverable damage under higher PFA exposures and a reduced likelihood of posttreatment recoverability (e.g., via sublethal injury and/or regrowth) as ICT increases (Maffettone et al. 2020). Such outcomes are favorable for AMR control, as reduced recoverability limits opportunities for resistant enterococci to persist or regrow.

The observed shift in PFA kinetic breakpoints between standard and extended incubation is consistent with exposure‐dependent sublethal injury and/or recoverable states at lower ICTs. However, higher ICTs are required to sustain equivalent inactivation under extended incubation, consistent with the induction of sublethal oxidative stress at lower ICTs and improved durability at higher exposures. This aligns with recent studies (Yin et al. 2023) suggesting that PAA can induce antibiotic‐resistant *E. coli* into a VBNC state. As the smallest percarboxylic acid, PFA is partially protonated near neutral pH (logK ≈ 7.58 at 25°C), and peroxyacids have been observed to penetrate intact bacterial membranes and induce intracellular oxidative stress (Pellegrino et al. 2026; Wang et al. 2023). PFA can therefore access intracellular targets without necessarily causing immediate, gross cell‐envelope lysis during early exposure (Wang et al. 2023). In wastewater, PFA inactivation pathways include both direct and radical‐mediated routes; for *E. coli* , direct PFA reactions were estimated to contribute ~73% of inactivation, with •OH and peroxide radicals contributing ~20% and ~6%, respectively (Ding et al. 2023). PFA primarily oxidizes sulfur‐containing compounds and amines, disrupting essential cellular enzymes through oxygen‐transfer reactions (Nabintu Kajoka et al. 2025; Wang et al. 2023). This impairs adenosine triphosphate (ATP) generation, protein synthesis, and ion gradient maintenance, resulting in metabolic shutdown that prevents binary fission and renders cells nonculturable on standard media, potentially maintaining metabolic activity and structural integrity if ICT is not lethal; however, these attributes would require complementary viability assays for confirmation.

Reactivation/regrowth was observed for both TE and VRE in PAA experiments. Notably, the overall extent of inactivation achieved for PAA was lower than that for PFA. For example, seven instances with log_10_ reduction exceeding 2 occurred for PFA (TE under standard incubation), three of which corresponded to negligible reactivation, compared with only one instance for PAA. Accordingly, the PAA data remained concentrated within the recoverable range and did not extend sufficiently into the high‐inactivation region needed to evaluate whether a similar decline in recoverability would occur at higher exposure.

The microbial reactivation under extended incubation, particularly for VRE (Figures 4 and 3a,b), is consistent with prior studies indicating that PAA can be associated with VBNC formation and/or post treatment regrowth. This differential response between standard and extended incubation poses a potential public health concern, as standard incubation protocols might overestimate PAA efficacy (Truchado et al. 2021).

**FIGURE 5:**
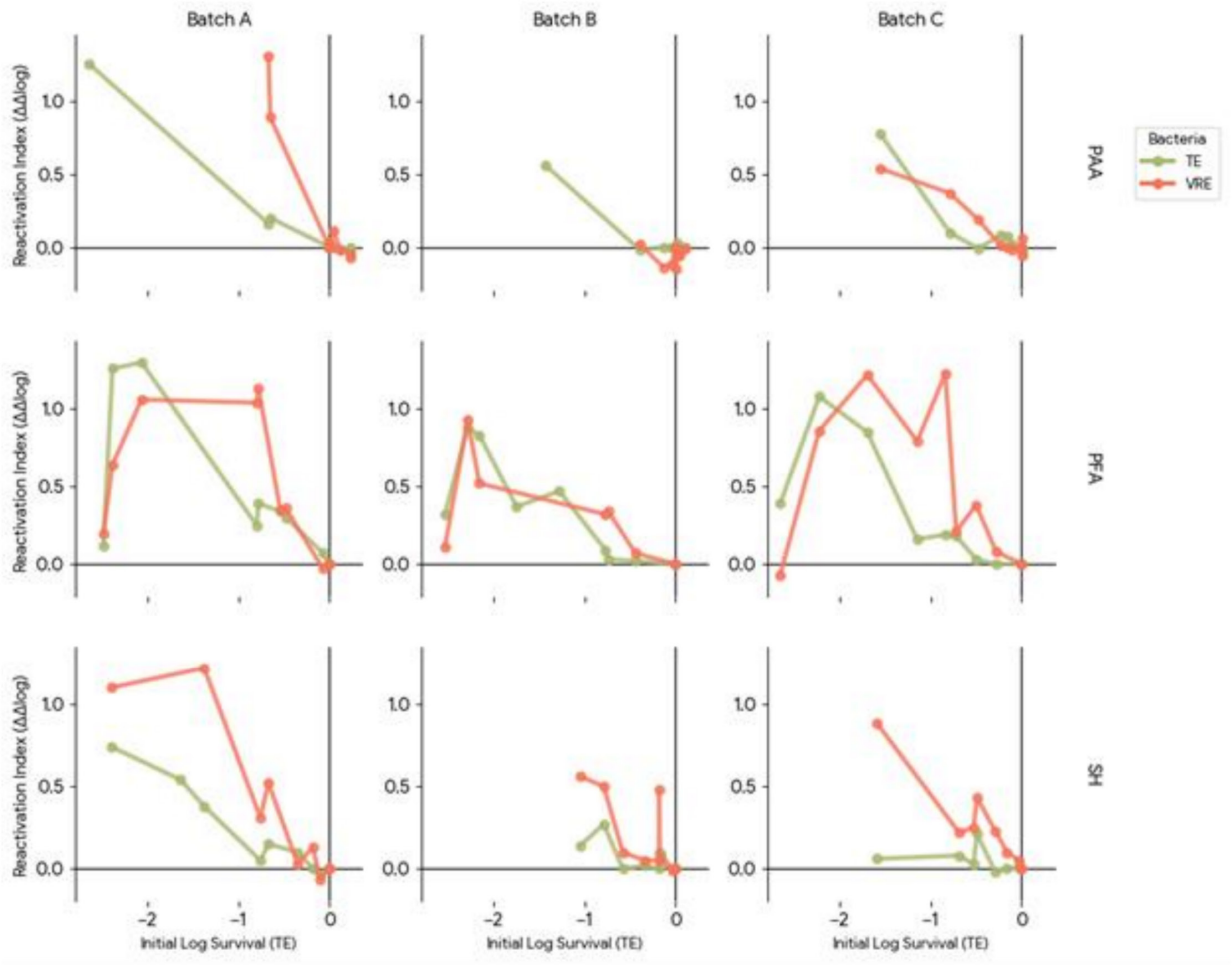
Reactivation index (RI) for TE and VRE plotted against standard‐incubation TE log_10_ reduction for PFA, PAA, and NaOCl across experimental batches (A–C). NaOCl, sodium hypochlorite; PAA, peracetic acid; PFA, performic acid; RI, reactivation index; TE, total enterococci; VRE, vancomycin‐resistant enterococci.

The RI analysis (Figure 5), which isolates the net increase in viable cells attributable to biological reactivation rather than methodological bias due to potential inhibitor breakdown during extended incubation, reveals critical findings. It indicates a critical dependency between the magnitude of immediate damage and the capacity for bacterial repair. Normalizing reactivation against the initial log reduction minimizes bias in evaluating whether a disinfection process achieves permanent kill or results in potentially reversible injury states (including VBNC injury) and/or regrowth, within the constraints of culture‐based measurement.

For PFA, conditions achieving higher initial inactivation (> ~2.5 log reduction) were associated with negligible RI values. This pattern supports the presence of an operational durability threshold, whereby increasing exposure and achieved inactivation correspond to negligible recoverability, indicating robust inactivation under the tested conditions.

In contrast, the data for PAA and NaOCl are currently limited by a “range bias.” In most replicates (specifically Batches B and C), the maximum inactivation achieved by PAA and NaOCl was substantially lower than for PFA. Consequently, data for these disinfectants cluster in the “reversible zone,” where cellular damage is insufficient to prevent repair, resulting in high RI (RI > 0). This disparity limits direct comparison across disinfectants and suggests that conclusions regarding relative performance should be bounded to the tested ICT ranges. These results do not establish the intrinsic superiority of one disinfectant but rather indicate that PAA and NaOCl were not evaluated at sufficiently high exposure conditions to assess whether similar reductions in recoverability could be achieved.

The benchmarking of VRE using ICT and the RI provides a meaningful framework for addressing the US EPA's 2025 emphasis on “exposure durability,” extending evaluation beyond immediate inactivation to include suppression of posttreatment recoverability. Identification of an operational durability threshold for PFA offers a practical indicator for defining exposure conditions associated with minimal recoverability. Within the studied exposure range (28–34 mg·min/L ICT), RI approached negligible levels under the tested conditions, supporting the use of ICT‐based criteria for designing robust disinfection strategies. This framework is particularly relevant for risk‐based engineering and wastewater reuse applications.

This framework provides a bridge between conventional log‐reduction targets and risk‐based performance metrics used in quantitative microbial risk assessment (QMRA). By linking ICT thresholds to suppression of reactivation, these findings support the development of exposure‐based criteria that better reflect durable pathogen inactivation and reduced risk of downstream transmission.

The formation of VBNC cells is a critical public health concern because they may retain virulence, harbor, and transfer resistance genes, contributing to the environmental dissemination of AMR despite being nonculturable by standard methods (Prosdocimi et al. 2023). This phenomenon risks unintended release of AMR pathogens into receiving environments or water reuse applications, contributing to the broader environmental dimension of AMR (Hart et al. 2023; Summerlin et al. 2021). Chlorination (or chloramination) at higher doses exacerbates the risk by destroying cell walls and oxidizing cellular materials without degrading robust ARGs like *vanA*. As a result, intracellular deoxyribonucleic acid (DNA) is released into the environment, where extracellular DNA (eDNA) can reach concentrations 25 times greater than intracellular DNA. The promotion of ARG exchange by chlorine allows surviving viable bacteria to acquire this persistent genetic material through HGT or transformation, effectively increasing the resistance profile of the remaining microbial community (Furukawa et al. 2015). In contrast to chlorination, the “penetrate‐and‐neutralize” mechanism of peroxyacids like PFA and PAA exhibits “promising potential in controlling cell lysis and ARG release,” thereby offering a dual advantage for ARB control in secondary effluent: high biocidal potency and superior containment of the genetic precursors for environmental antibiotic resistance (M. Wang et al. 2022).

The extended incubation step used in this study provides insight into posttreatment recovery of culturability (reactivation/regrowth) in water systems (Nguyen et al. 2017). However, because culturability‐based recovery can reflect either VBNC resuscitation or regrowth of residual culturable cells, interpretation shares established methodological considerations common to the field (L. Li et al. 2014; Shenghua Zhang et al. 2018; Zhao et al. 2017). Accordingly, the RI should be interpreted as an apparent reactivation metric reflecting net changes in culturability rather than a definitive measure of VBNC resuscitation. Complementary methods such as pre‐rRNA with nutritional stimulation (Alfahham et al. 2025), PMA‐qPCR, or transcriptomic analysis would strengthen inference about recovery mechanisms.

These considerations highlight opportunities for methodological refinement, enhancing the robustness of reactivation assessments without diminishing the demonstrated threshold effects and disinfectant performance insights. Even if results may be impacted by growth of the population not inactivated during extended incubation, this approach still identifies disinfectants, such as PFA, capable of providing both the best inactivation of the initial population and minimizing posttreatment recoverability consistent with VBNC injury and/or regrowth.

Although this study focused exclusively on culture‐based inactivation endpoints and did not quantify ARGs, the critical role of ARG abundance and HGT in AMR propagation in wastewater underscores the need for future work to evaluate ARG persistence and potential reductions following PFA, PAA, and NaOCl treatment, with particular emphasis on VRE‐associated resistance determinants.

In summary, this study demonstrates that microbial inactivation assay choice affects observed disinfection outcomes and interpretations. The standard and extended incubation protocols for assessing inactivation and recovery of both total and resistant populations provide a framework for further work to evaluate the public health relevance of residual VBNC populations and resistant fractions. Although results show differences in observed posttreatment recoveries, they do not provide a definitive comparative ranking for the reactivation potential of the disinfectants across all operational conditions. Targeted experiments and risk‐based assessments will be needed to translate these findings into operational guidance.

## 5 Conclusions, Recommendations, and Future Work

This study demonstrates that PFA provides the most reliable and predictable disinfection performance at the doses evaluated among the tested disinfectants, exhibiting rapid, largely linear inactivation under the experimental conditions evaluated. PFA acted nonselectively, with VRE and TE displaying statistically similar inactivation kinetics, consistent with radical‐mediated disinfection being generally AMR‐nonselective (Azuma et al. 2023). Although limited reactivation was observed at low ICT exposures, reactivation became negligible at ICT > 28 mg·min/L, indicating sustained inactivation and a low propensity for inducing VBNC states at higher exposures.

PAA did not exhibit evidence of ARB selection but showed slower, more variable inactivation kinetics with pronounced shouldering, frequently requiring higher ICT values to achieve comparable TE and VRE reductions. NaOCl exhibited the most complex and least reliable behavior, characterized by inconsistent inactivation and nonmonotonic dose–response relationships.

Within the ICT range evaluated, PFA most consistently achieved durable inactivation resulting in negligible recovery above ~28–34 mg·min/L; however, PAA and NaOCl were not tested at matched high‐LR exposures; therefore, cross‐oxidant durability thresholds cannot yet be compared definitively. Nonetheless, results reveal a clear threshold effect whereby disinfection permanence depends on the extent of initial inactivation achieved. RI values for PFA collapsed to near‐zero once log reductions exceeded a critical threshold, suggesting that sufficient ICT can overwhelm bacterial repair mechanisms and prevent recovery. Because this threshold was not reached for PAA and NaOCl within the tested ICT range, reactivation and VBNC persisted for both TE and VRE.

In addition to minimizing concerns associated with disinfection by‐product formation, PFA offers operational advantages. It contributes substantially less organic load to the effluent compared with PAA, which is operationally meaningful for utilities operating near COD discharge limits or seeking to minimize downstream dechlorination and ecological toxicity risks. Together, these attributes position PFA as a compelling alternative for AMR‐conscious wastewater disinfection.

By integrating ICT exposure with AMR selectivity and reactivation metrics, this study establishes a practical and transferable framework for benchmarking ARB disinfection and identifying exposure thresholds that suppress posttreatment recovery. This approach supports more risk‐informed decision‐making for utilities evaluating alternative disinfectants. The findings further underscore the need to reassess PAA and NaOCl at higher ICT ranges and across diverse wastewater matrices to determine whether irreversible inactivation thresholds comparable to those observed for PFA can be achieved. To support translation of these findings into practice, a conceptual decision framework is proposed. In ammonia‐rich secondary effluents where chloramination dominates and ARB control is a priority, disinfectants capable of achieving ICT thresholds associated with minimal reactivation (e.g., > 28 mg·min/L for PFA under tested conditions) may offer advantages over conventional approaches. In contrast, systems constrained by chemical handling or generation requirements may require optimization of PAA or chloramine‐based strategies to achieve comparable durable inactivation. This framing highlights the need to align disinfectant selection with both water quality conditions and treatment objectives (Figure S7).

### 5.1 Practical and Engineering Implications for ARB Control

Based on the findings, the following are implications for VRE control, sustained TE suppression, and avoidance of selective pressure:

- Consider PFA for ARB‐conscious disinfection: Utilities prioritizing AMR and ecosystem protection should evaluate PFA as an opportunity to obtain high efficacy that minimizes secondary environmental risks. Although PFA requires on‐site generation and precise dosing due to rapid decay, the absence of persistent residuals may eliminate the need for a removal step, providing an operational advantage over NaOCl, which typically requires dechlorination to protect sensitive aquatic species.
- Re‐evaluate PAA and NaOCl exposure requirements: The ICT required for effective VRE control using PAA and NaOCl should be critically reassessed. Comparative studies using matched‐exposure designs that target equivalent log‐reduction endpoints across disinfectants are needed to enable unbiased reactivation assessment.
- Move beyond CT‐based compliance alone: Disinfection design and monitoring should explicitly account for microbial reactivation and VBNC formation. Reliance on standard CT‐based compliance may underestimate viable pathogen persistence, particularly for resilient organisms such as VRE. For NaOCl in ammonia‐rich secondary effluents, longer contact times rather than higher doses should be prioritized, leveraging slower decay to achieve target reductions while limiting chemical use and disinfection by‐product formation.
- Incorporate long‐term viability assessment: Because all disinfectants can induce VBNC states that evade routine culture‐based detection, utilities are encouraged to supplement standard assays with extended incubation or molecular viability methods, especially when effluent is discharged to sensitive receiving waters or reused for irrigation or indirect potable applications.
- Implementation considerations remain an important factor in disinfectant selection. Although PFA demonstrates favorable performance characteristics, its requirement for on‐site generation, handling of concentrated precursors, and associated safety protocols may influence feasibility at full scale. In contrast, PAA and chlorination systems benefit from established supply chains and operational familiarity but may require higher exposures or additional controls to achieve comparable durability. A comparative evaluation of cost, safety, and operational complexity is warranted to support full‐scale adoption.

Future research should validate these findings across diverse full‐scale wastewater matrices, elucidate VRE recovery mechanisms through genetic and transcriptomic analyses, and refine ICT modeling to better resolve reactivation thresholds. Additional efforts should incorporate kinetic frameworks that jointly estimate initial disinfectant concentration and decay rates, evaluate impacts on ARG abundance and HGT potential, and link laboratory outcomes with QMRA. Integrating disinfection performance with operational considerations, including cost, safety, and system complexity, will further support translation to full‐scale practice.

### 5.2 Supporting Information

Supporting Information includes (1) preliminary AMR assessment in WRRF secondary effluent; (2) modified IDEXX protocol for antibiotic‐resistance quantification in wastewater; (3) bench‐scale disinfection assessment protocol; (4) disinfectant decay in the tested samples; (5) the complex interplay between disinfectant dose, efficacy, and the physicochemical properties of the wastewater matrix; (6) COD increase ratio framework: PAA versus PFA; and (7) disinfectant selection decision tree.

## 6 Limitations

This investigation employed controlled laboratory conditions, focusing on a limited selection of disinfectants and enterococci strains. The use of effluent from a single WWTP is crucial for minimizing confounding variables and establishing rigorous initial comparisons, and results in findings that offer foundational data. However, this focused approach does not fully capture the extensive physical, chemical, and biological variability in diverse full‐scale WWTPs. Factors such as seasonal and site‐specific variations in water hardness, pH, temperature, ammonia, suspended solids, and organic matter composition can influence disinfectant efficacy and microbial responses.

The findings are specific to the ICT range, incubation conditions, secondary wastewater effluent characteristics, disinfectant concentrations, contact times, and microbial analysis methods used. Although reproducibility was a priority, the inherent variability in complex wastewater matrices and biological systems can influence outcomes.

A conceptual limitation is that ICT values are disinfectant‐specific; thus, interpreting disinfectant performance based solely on ICT requires caution. ICT values are not interchangeable among disinfectants, as each has unique chemistry that dictates different dose–response relationships and cellular targets. An equivalent ICT for different disinfectants can lead to varied microbial outcomes, including irreversible damage versus sublethal injury or VBNC states, profoundly influencing microbial injury and recovery dynamics.

Despite these limitations, the results provide valuable insights into the comparative performance of these disinfectants under controlled conditions. These findings establish a foundation for future research to expand their applicability to broader contexts.

## Author Contributions

**Nuha Alfahham:** conceptualization, investigation, writing – original draft, methodology, validation, visualization, writing – review and editing, software, formal analysis, data curation. **Katherine Y. Bell:** conceptualization, funding acquisition, writing – review and editing, resources, methodology. **Reem Suleiman:** writing – original draft, formal analysis, data curation. **John Norton:** writing – review and editing, resources, funding acquisition, conceptualization. **Glen T. Daigger:** conceptualization, investigation, funding acquisition, methodology, validation, visualization, writing – review and editing, software, formal analysis, project administration, supervision, resources.

## Funding

This work was funded by the Great Lakes Water Authority (GLWA) through grant no. N038217.

## Ethics Statement

Ethics approval was not required because no human participants, identifiable data, or animals were involved. Wastewater samples and cultures were handled in accordance with institutional biosafety procedures for environmental microbiology, including appropriate PPE and decontamination/disposal of biological waste.

## Consent

The authors have nothing to report.

## Conflicts of Interest

The authors declare no conflicts of interest.

## Supporting information


**Figure S1:** Workflow for assessing tetracycline, vancomycin, and amp resistance in *Escherichia coli* and enterococci in secondary wastewater effluent using parallel sample aliquots, selective antibiotic amendment, and enumeration with Colilert and Enterolert systems.
**Figure S2:** Antibiotic resistance assessment of Escherichia coli and enterococci in secondary wastewater effluent samples from the WRRF at Great Lakes Water Authority (GLWA). This bar chart displays the percentage of resistance observed for various combinations of antibiotics and bacterial groups. The ‘Sample ID’ categories on the x‐axis represent the specific antibiotic tested, Vancomycin, Ampicillin, or Tetracycline, and the bacterial group, enterococci or Escherichia coli. The y‐axis indicates the percentage of bacterial resistance.
**Table S1:** Stoichiometric evaluation of sodium thiosulfate capacity in IDEXX sampling vessels relative to oxidant demand from peroxyacids. Calculations are based on the manufacturer‐specified ability of the vessels to neutralize at least 15‐mg/L chlorine in a 100‐mL sample and a conservative 2:1 thiosulfate‐to‐oxidant molar demand. Excess factors represent the ratio of available thiosulfate to the theoretical requirement for the tested concentrations of PFA and PAA.
**Figure S3:** Residual decay profiles of free chlorine (FC) and total chlorine (TC) across distinct wastewater matrices over a 30‐min contact window: (a) FC residual at an initial dose of 2.0 mg/L; (b) FC residual at an initial dose of 4.0 mg/L; (c) TC residual at an initial dose of 2.0 mg/L; and (d) TC residual at an initial dose of 4.0 mg/L. Markers differentiate independent wastewater batches (Replicates A, B, and C).
**Figure S4:** Peracetic acid (PAA) residual consumption kinetics under varying applied demands in municipal wastewater: (a) PAA residual following an initial applied dose of 2.0 mg/L; and (b) PAA residual following an initial applied dose of 4.0 mg/L. Solid lines represent the progressive depletion tracking across three discrete wastewater batches (Replicates A, B, and C) over 30 min of contact time.
**Figure S5:** Performic acid (PFA) residual dissipation profiles under low and moderate initial dosing configurations: (a) PFA residual tracking an initial applied dose of 1.0 mg/L; and (b) PFA residual tracking an initial applied dose of 2.0 mg/L. Stratified symbols denote concentration tracking across independent wastewater matrices (Replicates A, B, and C) within a 30‐min contact window.
**Table S2:** Statistical significance of factors from ANCOVA interaction models (Log_10_ Reduction ~ ICT × Replicate_ID × Dose_mg/L × Incubation_Type × Bacteria_Type) on disinfectant efficacy. Significant (p < 0.05); no effect (p ≥ 0.05). NaOCl, sodium hypochlorite; PAA, peracetic acid; PFA, peracetic acid–formic acid.
**Figure S6:** Exploratory correlation analysis between wastewater matrix (WWQ) parameters and disinfection performance metrics for PFA, PAA, and NaOCl (as TC). Pearson correlation coefficients relating measured water quality parameters to observed disinfection responses across experimental batches were not statistically significant (p > 0.05). Analysis was performed using the linear inactivation range.
**Table S3:** Literature summary of comparative disinfection efficacies for peracetic acid–formic acid, PAA, and NaOCl in wastewater treatment effluents. Reported ICT or Concentration × Time (CT) values (primarily in mg·min/L) denote exposures required for ~3–4 log_10_ reductions of target bacteria (e.g., E. coli and enterococci), derived from linear inactivation models within validated ranges; key notes highlight methodological caveats and matrix specifics.
**Table S4:** PAA compositions are taken from Peragreen 22 and Peragreen 15 technical data sheets (Enviro Tech Chemical Services Inc., n.d.), the VigorOx WWT II formulation description (PeroxyChem 2014), and the Proxitane WW‐12 MSDS (Solvay Chemicals Inc. 2003). PFA bounds use the disclosed 10‐ to 16‐wt% active‐PFA range and the presence of unreacted FA and H2O2 in the equilibrium mixture (Aubeuf‐Prieur et al. 2021). The H2O2 interference coefficient (0.4706) follows the dichromate COD interference correction described by Kang et al. (1999). Sources for PAA cases: Peragreen 22 TDS ranges, Peragreen 15 TDS ranges, VigorOx WWT II digest, Proxitane WW‐12 MSDS.
**Figure S7:** Conceptual operational decision tree for disinfectant selection using ICT–reactivation evidence. Note: Cost/DBP/COD‐related cues in the figure are included only to support feasibility screening and should be evaluated using site‐specific design constraints and regulatory context during local validation. Cost and DBP factors are conceptual placeholders outside the scope of this study.

## Data Availability

Relevant data are included in the paper or its Supporting Information.
