# Supplementary material for: Toward Robust ARB Inactivation in Wastewater: Comparing Performic Acid, Peracetic Acid, and Chloramination Using ICT, Resistance, and Reactivation Metrics
Source: Water Environ Res. 2026 Jul 5;98(7):e70461. doi: 10.1002/wer.70461 (PMC13333321; doi:10.1002/wer.70461)
Supplement: Supplementary file 1 — Figure S1: Workflow for assessing tetracycline, vancomycin, and amp resistance in Escherichia coli and enterococci in secondary wastewater effluent using parallel sample aliquots, selective antibiotic amendment, and enumeration with Colilert and Enterolert systems. Figure S2: Antibiotic resistance assessment of Escherichia coli and enterococci in secondary wastewater effluent samples from the WRRF at Great Lakes Water Authority (GLWA). This bar chart displays the percentage of resistance observed for various combinations of antibiotics and bacterial groups. The ‘Sample ID’ categories on the x‐axis represent the specific antibiotic tested, Vancomycin, Ampicillin, or Tetracycline, and the bacterial group, enterococci or Escherichia coli. The y‐axis indicates the percentage of bacterial resistance. Table S1: Stoichiometric evaluation of sodium thiosulfate capacity in IDEXX sampling vessels relative to oxidant demand from peroxyacids. Calculations are based on the manufacturer‐specified ability of the vessels to neutralize at least 15‐mg/L chlorine in a 100‐mL sample and a conservative 2:1 thiosulfate‐to‐oxidant molar demand. Excess factors represent the ratio of available thiosulfate to the theoretical requirement for the tested concentrations of PFA and PAA. Figure S3: Residual decay profiles of free chlorine (FC) and total chlorine (TC) across distinct wastewater matrices over a 30‐min contact window: (a) FC residual at an initial dose of 2.0 mg/L; (b) FC residual at an initial dose of 4.0 mg/L; (c) TC residual at an initial dose of 2.0 mg/L; and (d) TC residual at an initial dose of 4.0 mg/L. Markers differentiate independent wastewater batches (Replicates A, B, and C). Figure S4: Peracetic acid (PAA) residual consumption kinetics under varying applied demands in municipal wastewater: (a) PAA residual following an initial applied dose of 2.0 mg/L; and (b) PAA residual following an initial applied dose of 4.0 mg/L. Solid lines represent the progressive depletion trac [file WER-98-e70461-s001.docx]

# Supplementarty Information Towards Robust ARB Inactivation in Wastewater: Comparing Performic Acid, Peracetic Acid, and Chloramination using ICT, Resistance, and Reactivation Metrics

Nuha Alfahham ^1,2^, Katherine Y Bell ^3^, Reem Suleiman ^4^, John Norton ^5^, Glen T Daigger ^6^*

^1^Department of Civil and Environmental Engineering, University of Michigan, Ann Arbor, MI, USA.

^2^Brown and Caldwell, Troy, MI, USA.

^3^Hazen and Sawyer, Nashville, TN, USA.

^4^Department of Biomedical Engineering, University of Michigan, Ann Arbor, MI, USA.

^5^Great Lakes Water Authority, Detroit, MI, USA.

^6^*Department of Civil and Environmental Engineering, University of Michigan, Ann Arbor, MI, USA.

# List of Abbreviations

| **Acronym** | **Definition** |
| --- | --- |
| AMR | Antimicrobial Resistance |
| ANCOVA | Analysis of Covariance |
| ARB | Antibiotic-Resistant Bacteria |
| AOP | Advanced Oxidation Process (optional add-on) |
| BSL-1 | Biosafety Level 1 |
| CAPEX/OPEX | Capital/Operating Expenditure |
| CFU | Colony-Forming Units |
| CT | Concentration × Time (disinfection surrogate) |
| DBP(s) | Disinfection byproduct(s) |
| DI | Deionized Water |
| DPD | N,N-diethyl-p-phenylenediamine (colorimetric method for chlorine/chloramine) |
| FA | Formic Acid |
| FC | Free Chlorine |
| GLWA | Great Lakes Water Authority |
| H₂O_2_ | Hydrogen peroxide (present in PAA equilibrium mixtures and in PFA precursor/product mixtures) |
| H₂SO₄ | Sulfuric Acid |
| ICT | Integrated Concentration–Time (time-resolved exposure) |
| NaOCl | Sodium Hypochlorite |
| PAA | Peracetic Acid |
| PFA | Performic Acid |
| RI | Reactivation Index (ΔΔlog; extended vs. standard incubation) |
| SH | Sodium Hypochlorite |
| TC | Total Chlorine (preferred oxidant metric in ammonia rich matrices) |
| TE | Total Enterococci |
| ThOD | Theoretical oxygen demand (stoichiometric oxygen demand) |
| UV | Ultraviolet (disinfection) |
| VBNC | Viable but non-culturable |
| VRE | Vancomycin-Resistant Enterococci |
| WRRF | Water Resource Recovery Facility *(used interchangeably with WWTP)* |
| wt% | mass percent (w/w) |

Preliminary antimicrobial resistance assessment in Water Resource Recovery Facility (WRRF) Secondary Effluent

Standard EPA-approved Colilert (IDEXX, Catalog #WP200I) and Enterolert (IDEXX, Catalog #WENT200) test kits with the Quanti-Tray system (IDEXX, Catalog #WQT2K) for wastewater were utilized for the enumeration of Total Enterococci (TE) (Environmental Protection Agency, 2007). The IDEXX system offered several advantages over traditional plate-based methods, including reduced laboratory time, fewer required dilutions to achieve readings within the dynamic range, enhanced ease in maintaining sterility during analysis, and suitability for lower Biosafety Level 1 (BSL-1) conditions due to the contained nature of bacterial culturing within the Quanti-Trays, rendering it a more accessible tool for widespread monitoring.

For the preliminary antimicrobial resistance (AMR) assessment, indigenous total and antibiotic-resistant *E. coli* and Enterococci were quantified in each sample as described in Figure S1. Resistance to three antibiotics, ampicillin (Amp), tetracycline (Tet), and vancomycin (Van), was assessed in wastewater samples. Antibiotic resistance testing followed European Committee on Antimicrobial Susceptibility Testing (EUCAST) and United States Clinical and Laboratory Standards Institute (CLSI) resistance breakpoints and minimum inhibitory concentration (MIC) guidelines. Stock solutions of Amp in Milli-Q (32 mg/mL), Tet in 70% ethanol (16 mg/mL), and Van in Milli-Q (32 mg/mL) were prepared using Ampicillin Sodium Salt, Crystalline Powder (Fisher BioReagents™, Catalog # BP1760-25); Tetracycline Hydrochloride (MP Biomedicals, Catalog # ICN19454201); and Vancomycin Hydrochloride (MP Biomedicals, Inc., Catalog # ICN19554001), respectively. Required stock solution sterilization was achieved using 0.2 µm syringe filters (Fisher Scientific Basix Syringe Filters, MCE, Sterile, Catalog #13100102). The IDEXX Colilert and Enterolert Quanti-Tray methods were modified for assessing AMR based on previously validated protocols (Akiyama and Savin, 2010; Hornsby et al., 2023; Jimenez et al., 2025). Before sealing IDEXX Quanti-Trays, antibiotic stock solutions (220 µL of 32 mg/mL ampicillin (Amp), 420 µL of 16 mg/mL tetracycline, and 440 µL of 32 mg/mL vancomycin per 400 mL secondary effluent) were spiked directly into IDEXX sample bottles to achieve the desired final concentrations. Prepared solutions in IDEXX bottles were transferred to the Quanti-Tray and sealed using an IDEXX Quanti-Tray Sealer and incubated horizontally for 24-28 hours, at 35ºC ± 0.5ºC (*E. coli*), or 41°C ± 0.5°C (Enterococci). After incubation, trays were inspected under 365 nm ultraviolet (UV) illumination (6-Watt Fluorescent UV Lamp, IDEXX, Catalog #WL160) to quantify fluorescent wells indicating bacterial growth. Colony-Forming Units (CFU)/100 mL were determined using IDEXX MPN generator 1.4.4.


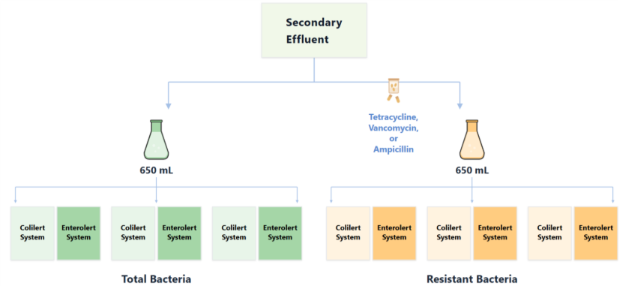


Figure S1. *Workflow for assessing tetracycline, vancomycin, and amp resistance in Escherichia coli and enterococci in secondary wastewater effluent using parallel sample aliquots, selective antibiotic amendment, and enumeration with Colilert and Enterolert systems.*

1. Modified IDEXX Protocol for Antibiotic-Resistance Quantification in Wastewater

Equipment

1. IDEXX Quanti-Tray Sealer

- 365-nm UV light (IDEXX 6 Watt UV Fluor Lamp, 110 V, Catalog #WL160)
- Autoclave
- Incubator

Reagents

- IDEXX Colilert and Enterolert test kits (Catalog #WP200I and #WENT200, IDEXX)
- Antibiotics: Ampicillin, Tetracycline, Vancomycin
- IDEXX bottles with sodium thiosulfate and bisulfate (IDEXX, Catalog #WV120SBST-200)
- DI Water
- Milli-Q purified water
- 0.2 µm syringe filters (Fisher Scientific Basix Syringe Filters, MCE, Sterile, Catalog #13100102)
- Ethyl alcohol, Pure 200 proof, ≥ 99.45%  (Sigma Aldrich Fine Chemicals Biosciences, Catalog # E7023-500)

Preparation of Antibiotics

- Prepare antibiotic solutions as follows:
- Ampicillin: Dissolve 64 mg in 2 mL sterile deionized water (DI) (32 mg/mL), sterilize using a 0.2 µm syringe filter, and store at -20ºC for up to six weeks.
- Tetracycline: Dissolve 32 mg in 2 mL 70% ethanol (16 mg/mL), sterilize using a 0.2 µm syringe filter, and store at -20ºC protected from light for up to two weeks.
- Vancomycin: Dissolve 64 mg in 2 mL sterile DI water (32 mg/mL), sterilize using a 0.2 µm syringe filter, and store at -20ºC for up to one year

Sample Collection and Preparation

- Collect wastewater samples in sterile, autoclaved carboys (Thermo Scientific, Catalog # 2197-0020).
- Partition samples into 1 L aliquots for experimental disinfection.
- Immediately transfer aliquots for COD, TSS, pH, and temperature analyses.
- Measure temperature and pH immediately using portable probes

Experimental Procedure

- Label IDEXX bottles and Quanti-Trays with date, time, disinfectant-antibiotic-dilution.
- Prepare dilutions in the IDEXX bottles as follows:
- 1X dilution: 100 mL secondary effluent (SE), no DI water.
- 10X dilution: 10 mL SE, 90 mL DI water.
- 20X dilution: 5 mL SE, 95 mL DI water.
- Add prepared antibiotic solutions directly into IDEXX bottles after wastewater addition, before transferring into Quanti-Trays.
- Add IDEXX Colilert or Enterolert powder to the bottles and mix until fully dissolved.
- Transfer contents into IDEXX Quanti-Trays and seal using IDEXX Quanti-Tray Sealer.
- Incubate trays horizontally, 24-28 hours, at 35ºC ± 0.5ºC (E. coli), or 41°C ± 0.5°C (Enterococci). Record the start and end times of incubation.
- According to IDEXX disposal guidelines, place used trays in double autoclave bags, ensuring the necks are loosely tied for steam ventilation. Set them in a suitable autoclavable tray or container, then autoclave at 121°C for 30–60 minutes at 15 psi on the liquid or biohazard waste cycle.

   Quantification of Total and Antibiotic-Resistant Bacteria (ARB)

- Following incubation, count fluorescent wells for total E coli. And Enterococci antibiotic-resistant populations under 365-nm UV light, following IDEXX protocols.


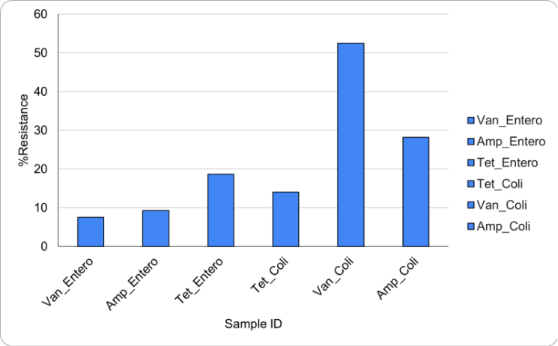


Figure S2. *Antibiotic resistance assessment of Escherichia coli and Enterococci in secondary wastewater effluent samples from the WRRF at Great Lakes Water Authority (GLWA). This bar chart displays the percentage of resistance observed for various combinations of antibiotics and bacterial groups. The 'Sample ID' categories on the x-axis represent the specific antibiotic tested, Vancomycin, Ampicillin, or Tetracycline, and the bacterial group, Enterococci or Escherichia coli. The y-axis indicates the percentage of bacterial resistance.*

1. Bench-Scale Disinfection Assessment Protocol

**Materials**

- Performic Acid (PFA) stock solution (~12-13 grams per 100 grams of total solution), prepared in lab
- Peracetic Acid (PAA) stock solution (~32 grams per 100 grams of total solution)
- Sodium Hypochlorite (NaOCl) stock solution (10-15% available chlorine)
- IDEXX 120 mL Quench Bottles (IDEXX 120 mL Shrink-Banded Vessels with Sodium Thiosulfate, Catalog #WV120SBST-200)
- Sterile deionized (DI) water
- Standard lab tools for liquid and solid handling (pipettes, micropipettes, graduated cylinders, beakers, magnetic stir plate and bars, timers, PPE)
- PAA SAM kit (CHEMetrics) for peracid/PFA concentration verification
- Hach DR900 or equivalent colorimeter for chlorine residual measurement

Procedure

- Preparation of Disinfectants
- PFA: Prepare by combining catalyzed formic acid (FA) (5 mL FA with 0.47 mL sulfuric acid (H₂SO₄)) with 5 mL hydrogen peroxide (35 wt%) at <15ºC. Allow to stabilize for about 90 minutes. Verify concentration using PAA SAM kit and convert to PFA by multiplying the reading by 0.816. Target stock concentration should be around 13 grams per 100 grams of total solution.
- PAA: Dilute 32% stock to target working concentration in DI water or wastewater (e.g., 6.25 µL per 1 L for 4 mg/L).
- NaOCl: Dilute 12-15 stock to target working concentration in DI water or wastewater (e.g., 33.3 µL per 1 L for 4 mg/L).
- Sample Setup
- Collect wastewater into sterile, autoclaved 10 L carboys.
- Partition into labeled 1 L aliquots for each disinfectant trial and for no-disinfection controls (one per disinfectant).
- Disinfection Contact Times
- Add disinfectant stock to achieve target experimental concentration (1-4 mg/L), start timer immediately while continuously mixing using a magnetic stir bar.
- Maintain constant mixing and collect aliquots at 0, 2, 5, 10, and 30 minutes.
- Pre-Quenching Measurement
- Measure residual disinfectant in each unquenched aliquot:
- Chlorine: N,N-diethyl-p-phenylenediamine (DPD) method (Hach DR900).
- PAA/PFA*: PAA SAM kit
- Quenching
- Immediately add the collected sample to the bottles preloaded with sodium thiosulfate/bisulfate for microbial tests
- Quench verification (confirming the absence of residual) was performed during validation and was not repeated for every subsequent run after validation.
- Post-Quenching
- Continue with microbial enumeration as described in the Modified IDEXX Protocol section (Supplementary Materials Appendix C-S2).

* The CHEMetrics DPD‑KI method is calibrated to report mg/L as peracetic acid (PAA). Because peracetic acid (PAA) and performic acid (PFA) are both peroxycarboxylic acids that liberate iodine with the same molar oxidizing equivalents (1 mol peracid produces 1 mol I₂), an instrument calibrated “as PAA” will report a PFA sample as mg/L as PAA.

To obtain the true mass concentration of PFA, the reported value should be multiplied by the molecular‑weight ratio: (Chemetrics guidance), as shown below:

PFA (mg/L) = Reading (mg/L as PAA)× 62.03/76.05 ​≈ Reading (mg/L as PAA) x 0.816

1. Quenching Capacity of Sodium Thiosulfate in Sample Vessels

#### Basis for Neutralization Capacity

IDEXX WV120SBST sampling vessels are specified to contain sufficient sodium thiosulfate (Na₂S₂O₃) to neutralize at least 15 mg/L residual chlorine in a 100 mL sample.

For a 100 mL sample:

- Mass of Cl₂:

$$15\text{ mg/L}\times0.1\text{ L}=1.5\text{ mg}=0.0015\text{ g}$$

Moles of Cl₂:

$$\frac{0.0015}{70.91}=2.12\times{10}^{-5}\text{ mol}\text{ }\text{Cl₂}$$

Using the standard dechlorination reaction (tetrathionate pathway):

$$2S_{2}O_{3}^{2-}+Cl_{2}\to S_{4}O_{6}^{2-}+2Cl^{-}$$

Required thiosulfate:

$$2 (2.12\times{10}^{-5})=4.23\times{10}^{-5}\text{ mol}\text{ }\text{thiosulfate}$$

- Equivalent mass (pentahydrate):

$$4.23\times{10}^{-5}\times248.18=10.5\text{ mg}$$

This is consistent with standard dechlorination practice (~7 mg Na₂S₂O₃·5H₂O per mg Cl₂).

#### Thiosulfate Demand for Peroxyacids

Peroxyacids (PFA, PAA) act as 2‑electron oxidants and are conservatively assumed to follow a 2:1 thiosulfate demand:

$$\text{Peroxyacid}+2S_{2}O_{3}^{2-}\to\text{Products}$$

**(a) PFA — 2 mg/L (100 mL sample)**

Mass:

$$2\times0.1=0.2\text{ mg}=0.0002\text{ g}$$

Moles:

$$\frac{0.0002}{62.03}=3.22\times{10}^{-6}\text{ mol}$$

Thiosulfate required:

##### $2 (3.22\times{10}^{-6})=6.44\times{10}^{-6}\text{ mol}$ thiosulfate

(b) PAA — 4 mg/L (100 mL sample)

Mass:

$$4\times0.1=0.4\text{ mg}=0.0004\text{ g}$$

Moles:

$$\frac{0.0004}{76.05}=5.26\times{10}^{-6}\text{ mol}$$

Thiosulfate required:

$$2 (5.26\times{10}^{-6})=1.05\times{10}^{-5}\text{ mol}\text{ thiosulfate}$$

#### Comparison to Available Capacity

Minimum thiosulfate available (based on chlorine neutralization spec):

$$4.23\times{10}^{-5}\text{ mol}$$

Table S1. Stoichiometric evaluation of sodium thiosulfate capacity in IDEXX sampling vessels relative to oxidant demand from peroxyacids. Calculations are based on the manufacturer-specified ability of the vessels to neutralize at least 15 mg/L chlorine in a 100 mL sample and a conservative 2:1 thiosulfate-to-oxidant molar demand. Excess factors represent the ratio of available thiosulfate to the theoretical requirement for the tested concentrations of PFA and PAA.

| **Oxidant** | **Required (mol)** | **Available (mol)** | **Excess** |
| --- | --- | --- | --- |
| mg/L PFA | $6.44\times{10}^{-6}$ | $4.23\times{10}^{-5}$ | **6.6×** |
| 4 mg/L PAA | $1.05\times{10}^{-5}$ | $4.23\times{10}^{-5}$ | **4.0×** |

1. Disinfectant Decay in the Tested Samples


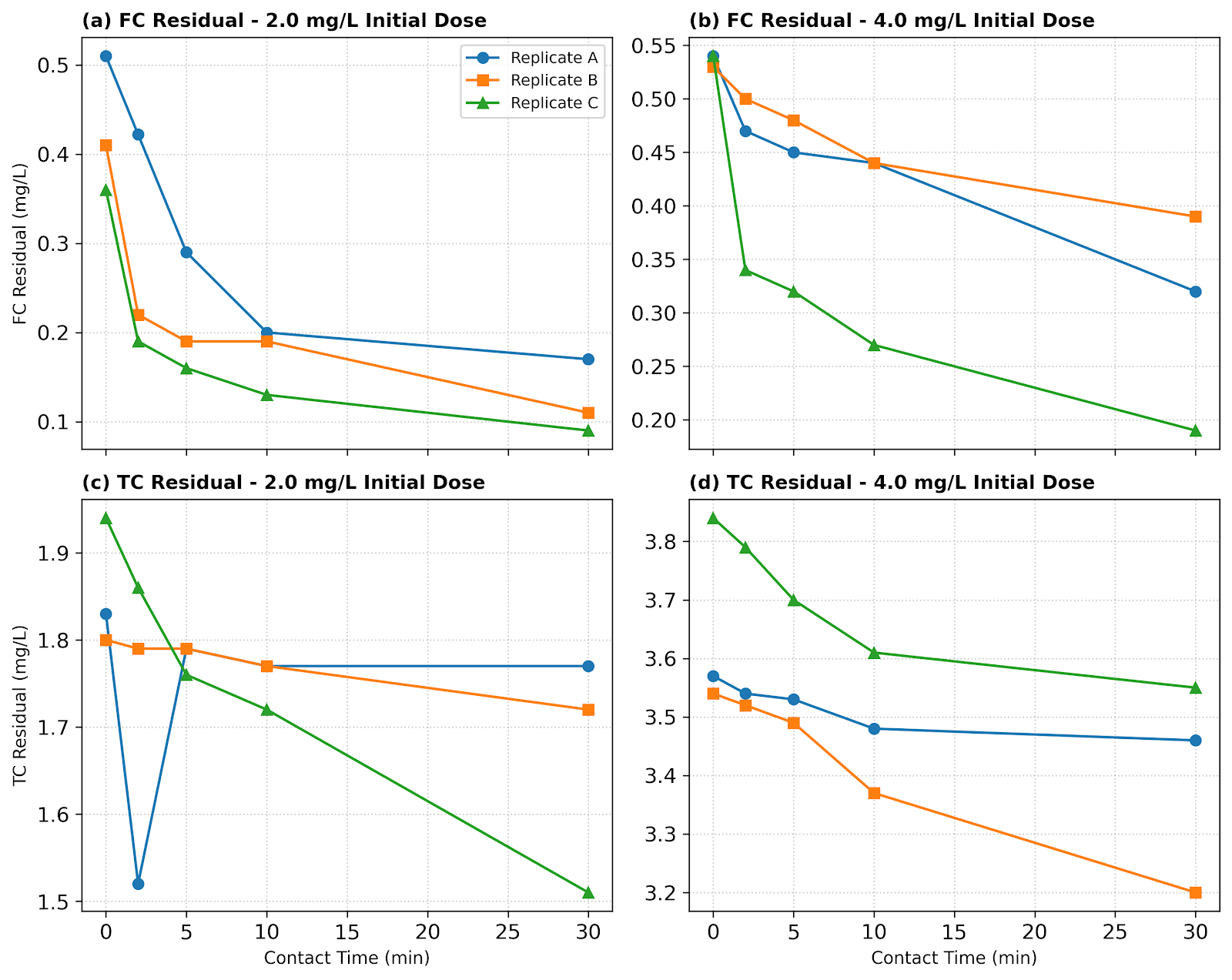


Figure S3. *Residual decay profiles of free chlorine (FC) and total chlorine (TC) across distinct wastewater matrices over a 30-min contact window: (a) FC residual at an initial dose of 2.0 mg/L; (b) FC residual at an initial dose of 4.0 mg/L; (c) TC residual at an initial dose of 2.0 mg/L; and (d) TC residual at an initial dose of 4.0 mg/L. Markers differentiate independent wastewater batches (Replicates A, B, and C).*


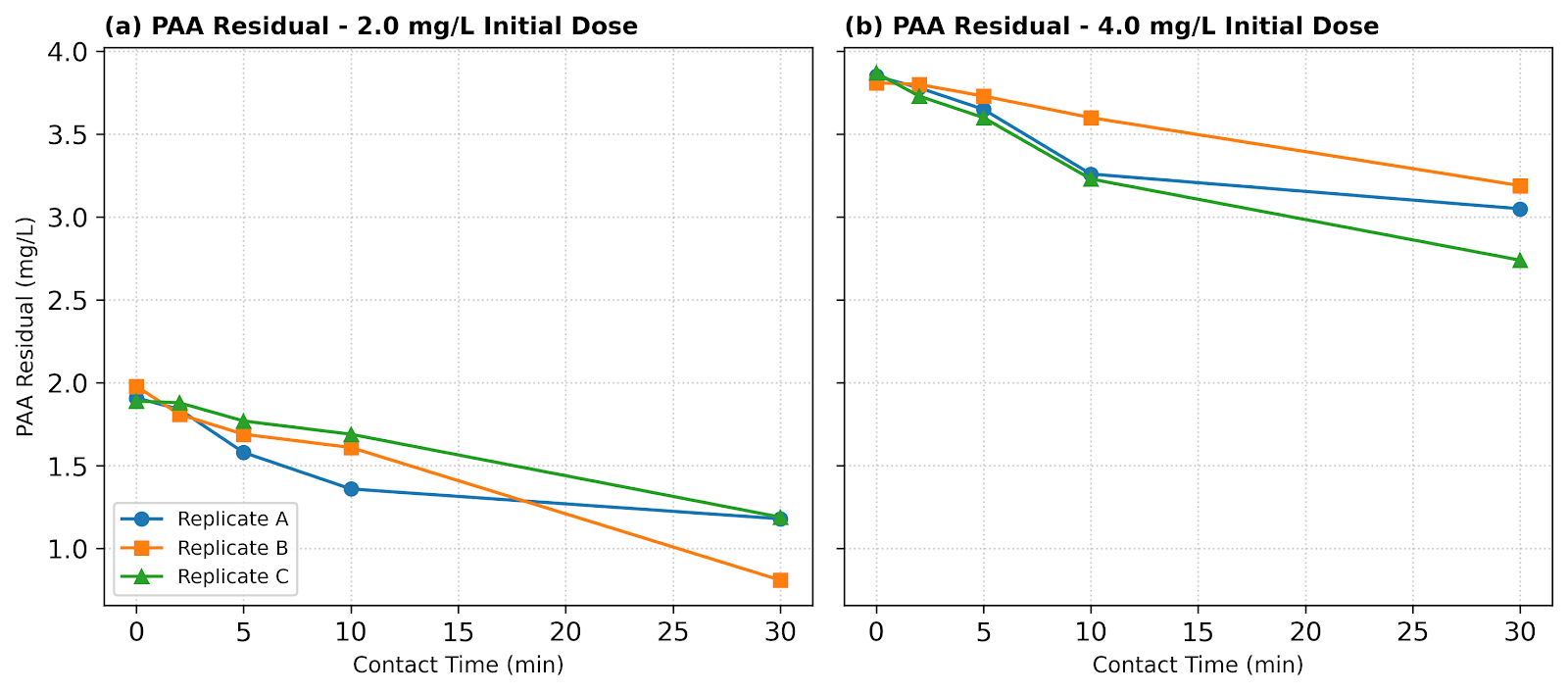


Figure S4. *Peracetic acid (PAA) residual consumption kinetics under varying applied demands in municipal wastewater: (a) PAA residual following an initial applied dose of 2.0 mg/L; and (b) PAA residual following an initial applied dose of 4.0 mg/L. Solid lines represent the progressive depletion tracking across three discrete wastewater batches (Replicates A, B, and C) over 30 min of contact time.*


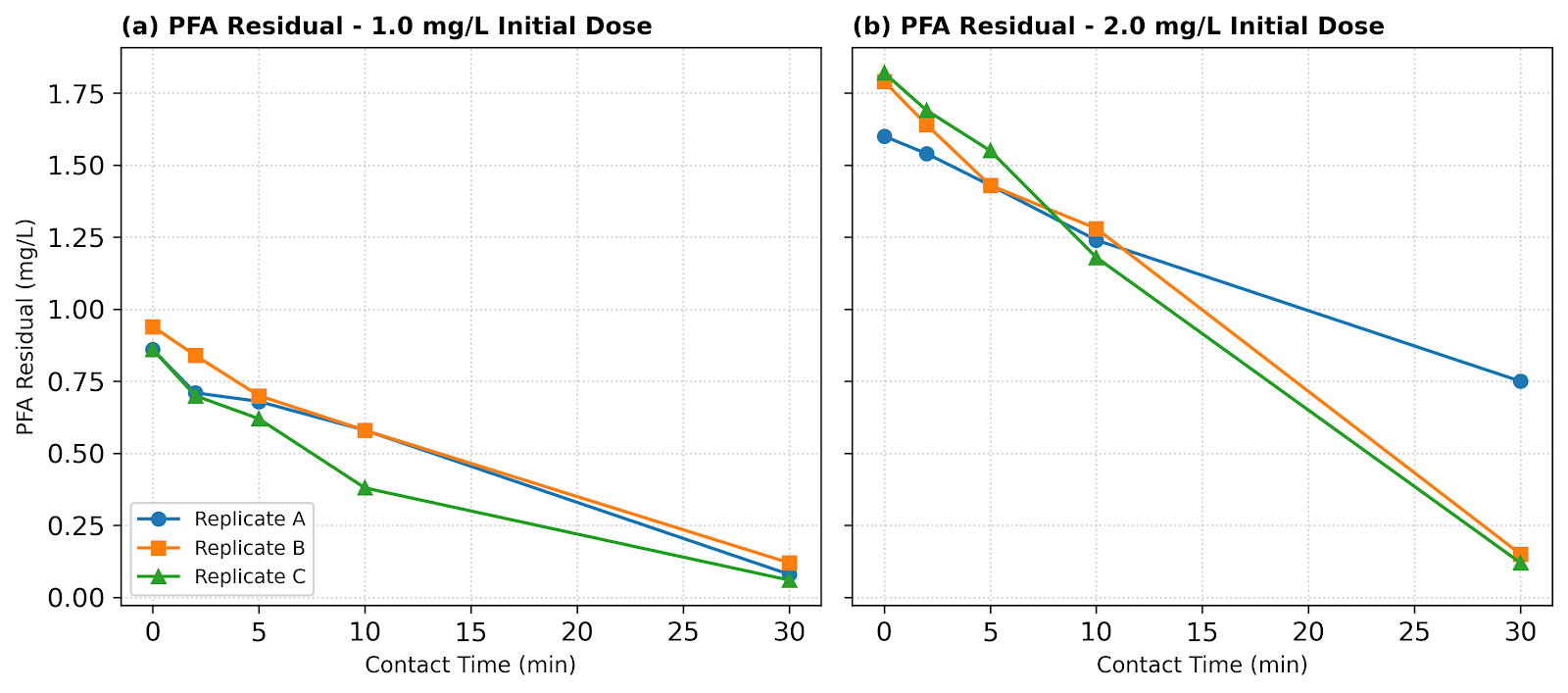


Figure S5. *Performic acid (PFA) residual dissipation profiles under low and moderate initial dosing configurations: (a) PFA residual tracking an initial applied dose of 1.0 mg/L; and (b) PFA residual tracking an initial applied dose of 2.0 mg/L. Stratified symbols denote concentration tracking across independent wastewater matrices (Replicates A, B, and C) within a 30-min contact window.*

1. The complex interplay between disinfectant dose, efficacy, and the physicochemical properties of the wastewater matrix

To analyze the factors influencing the *rate* of disinfection, a complex Analysis of Covariance (ANCOVA) was performed, mirroring the interaction model from Tableau. This model was run for each disinfectant (PFA, PAA, SH) on its definite linear inactivation range.  The model formula

Log₁₀ Reduction ~ Integrated Concentration x Time (ICT) (mg.min/L) * *C(Replicate_ID)  * C(Dose_mg_L) *  C(Incubation_Type)** C(Bacteria_Type)

This interaction-based (*) model tests for *sensitivity*. It evaluates how the kinetic relationship between ICT and Log₁₀ Reduction is altered by other factors, including the Replicate_ID, a proxy for the sample matrix.

1. PFA Results

For PFA, the disinfection kinetics exhibited high consistency across different experimental runs and concentrations. ANCOVA revealed that neither the applied dose (p = 0.675) nor the replicate ID (p = 0.256) significantly impacted the relationship between ICT and Log_10_ Reduction. Consequently, PFA data for all doses (1 and 2 mg/L) and all replicates (A, B, and C) were pooled into a single consolidated dataset for each combination of incubation and bacteria type. This grouping is statistically justified as the variation between replicates and doses did not exceed the inherent experimental noise. Within this pooled framework, Incubation Type remained the dominant driver of variance (p < 0.001), with Extended incubation consistently showing higher residual resistance compared to Standard incubation.

1. PAA Results

The analysis of PAA required stratification based on Replicate ID due to significant inter-run variability (p = 0.008). While the applied dose (2 and 4 mg/L) did not significantly alter inactivation efficiency (p = 0.846), the ANCOVA indicated that outcomes varied significantly between Replicates A, B, and C. This suggests that the PAA response was sensitive to daily fluctuations in the wastewater matrix. Therefore, while doses were grouped, the PAA results are presented as stratified by replicate. Notably, Bacteria Type (TE vs. Vancomycin-Resistant Enterococci (VRE)) was not a significant factor (p = 0.190), indicating a consistent oxidative response regardless of antibiotic resistance status.

1. TC Results

TC demonstrated the highest sensitivity to experimental variables, requiring granular stratification by both dose and replicate. ANCOVA confirmed significant effects for **Dose** (p = 0.041) and **Replicate ID** (p < 0.001).

While Bacteria Type was not significant in the aggregate model (p = 0.984), a more detailed sub-analysis revealed it was a significant factor in Replicates B (p = 0.018) and C (p = 0.048), particularly under Extended Incubation conditions. In these specific runs, VRE exhibited a different inactivation trajectory compared to TE, a finding that was masked in the global model by the lack of effect in Replicate A. This replicate-dependent sensitivity of the bacteria type effect, where antibiotic resistance may only confer a survival advantage under specific water quality or physiological conditions, further justifies the decision to present Cl-Total data without pooling, stratifying by dose, replicate, and incubation type to preserve these localized findings.

Table S2. Statistical significance of factors from ANCOVA interaction models (Log₁₀ Reduction ~ ICT × Replicate_ID × Dose_mg/L × Incubation_Type × Bacteria_Type) on disinfectant efficacy. Significant (p < 0.05); no effect (p ≥ 0.05). NaOCl, sodium hypochlorite; PAA, peracetic acid; PFA, peracetic acid-formic acid.

| Factor | NaOCl (Sodium Hypochlorite) | PAA (Peracetic Acid) | PFA (Performic Acid) |
| --- | --- | --- | --- |
| Incubation Type | S | S | S |
| Bacteria Type | S | NS | NS |
| Dose Group | S | NS | NS |
| Replicate ID | S | S | NS |

S = Significant, NS = Not significant


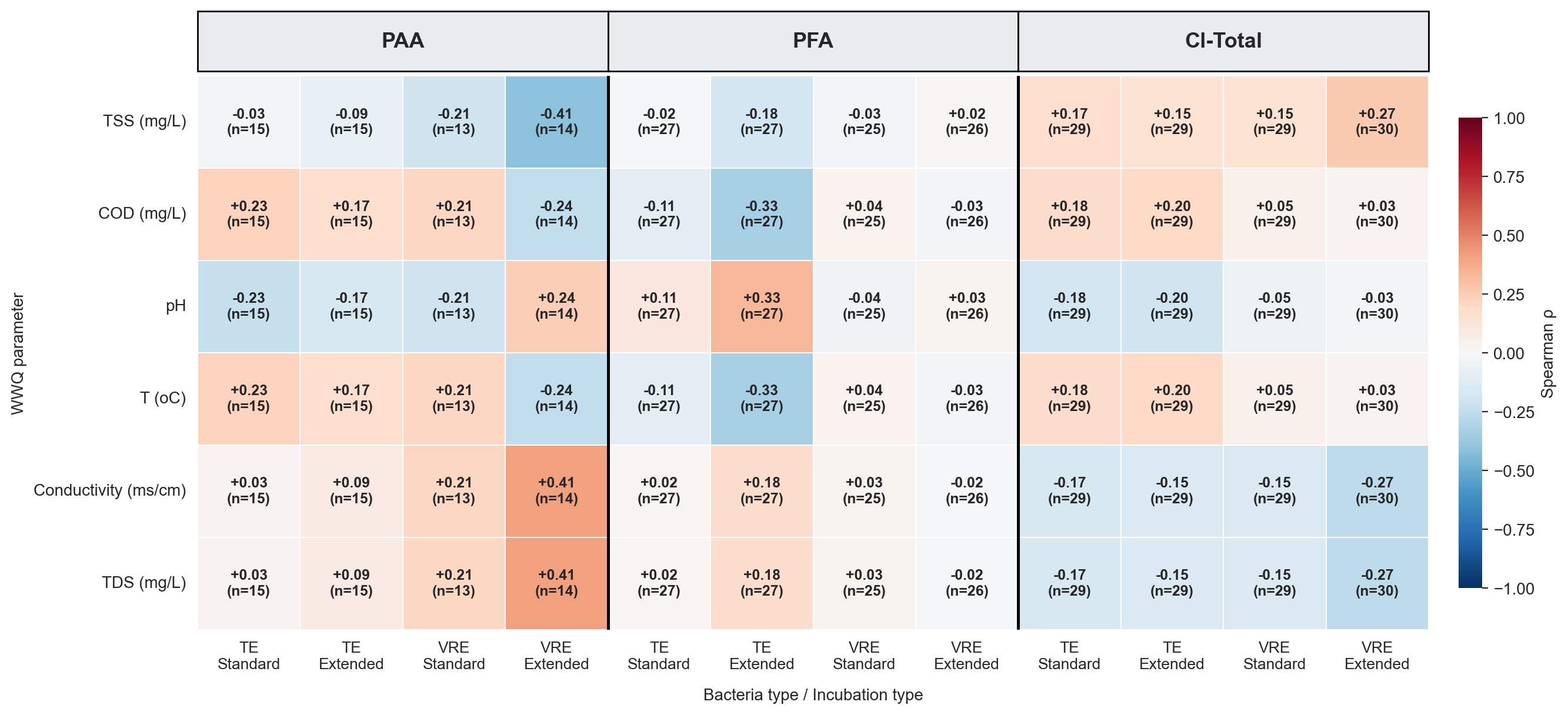


Figure S6. Exploratory correlation analysis between wastewater matrix (WWQ) parameters and disinfection performance metrics for PFA, PAA, and NaOCl (as TC). Pearson correlation coefficients relating measured water quality parameters to observed disinfection responses across experimental batches were not statistically significant (p > 0.05). Analysis was performed using the linear inactivation range.

NaOCl (TC)

Table S3. Literature summary of comparative disinfection efficacies for peracetic acid-formic acid, PAA, and NaOCl in wastewater treatment effluents. Reported ICT or Concentration x Time (CT) values (primarily in mg·min/L) denote exposures required for ~3-4 log₁₀ reductions of target bacteria (e.g., E. coli, enterococci), derived from linear inactivation models within validated ranges; key notes highlight methodological caveats and matrix specifics.

| Study | Disinfectants Compared | Matrix / Target Organisms | Comparative Framework | Key Effectiveness Findings (CT or ICT, mg·min/L) | Key Notes Relevant to Interpretation |
| --- | --- | --- | --- | --- | --- |
| **Ragazzo et al., 2020** | PFA, PAA, NaOCl (predominantly chloramines) | Secondary effluent; *E. coli*, enterococci | Range-based LR–ICT slopes within measured linear regions | PFA: ~20 (3-log *E. coli*); Cl: ~40–60; PAA: ~80–100 | Chlorine functioned mainly as monochloramine (>80%) due to background ammonia (0.1–10.3 mg/L NH₄–N); conclusions limited strictly to observed ICT windows |
| **Martínez-Campos, González-Pleiter, Fernández-Piñas, Rosal, & Leganés, 2021** | PFA vs PAA | Total and AR *E. coli* | Empirical LR–ICT slopes; equal-LR reading | PFA: ~10 (2-log), ~25 (3-log); PAA: ~25 (2-log), ~50 (3-log) | Steeper LR–ICT slope for PFA; comparisons based on fitted linear ranges, not isolated points |
| **Ocampo-Rodríguez et al., 2023** | NaOCl vs PAA | Wastewater isolates (*S. pasteuri*, *K. pneumoniae*, *B. subtilis*) | Target-LR CT normalization (1–3 log); kinetic model fits | NaOCl: ~3–7 to 419 (species-dependent, up to 4-log); PAA: ~20–287 | FC measured; no Enterococcus included; NaOCl generally required lower CT for Gram-negative targets |
| **Ding et al., 2023** | PFA vs NaOCl | Secondary effluent; temperature-dependent | LR vs CT/time across temperatures; regrowth tracking | PFA: ~15 (2-log, 25 °C); NaOCl: ~40 (2-log) | Strong post-disinfection regrowth after NaOCl; minimal regrowth after PFA; CT decreased ~30% at higher temperatures |
| **Sahulka et al., 2021** | Free Cl₂ vs PAA | *Enterococcus spp.* and *E. fergusonii* (resistant vs susceptible strains) | Strain-specific LR–dose/time curves; equal-LR comparison | Cl₂: ~100 (3-log); PAA: ~150 (3-log) | Focused on resistance phenotypes; no viable but non-culturable (VBNC) assessment; FC only |
| **Gehr, Chen, & Moreau, 2009** | (Gehr et al., 2009)PFA vs PAA (UV-context effluent) | Advanced primary effluent | Short-contact LR–time comparisons | PFA: ~153 (3-log); PAA: higher CT required for equivalent LR | Rapid-kill focus; conclusions based on clustered LR–time points rather than kinetic constants |
| **Bagagnan et al., 2024** | PFA | Effluent microbial community | LR and community-response ranges | Community impact plateau ≥60; peak damage ≈240 | Demonstrated strong disinfection with measurable but bounded community disruption |
| **Nyangaresi et al., 2026** | PFA, PAA,NaOCl | Multiple WRRF effluents | Range-wise LR–ICT families with uncertainty | PFA: ~15 (2-log); NaOCl: ~30; PAA: ~40 | Cross-plant consistency; conclusions based on slope distributions and overlapping ICT bands |

1. **COD Increase Ratio Framework: PAA vs. PFA**

Table S5 showing COD Increase Ratios for PAA vs. PFA was built from real product composition ranges for PAA and a bounded, stoichiometric framework for PFA using the 10–16 wt% active PFA generator-output range. All results are per mg active peracid delivered.

1. Symbols used **in the equations**

All computations are expressed **per mg of active peracid delivered** (i.e., normalized to 1 mg of active PAA or 1 mg of active PFA).

- $w_{p}$= mass fraction of **active peracid** in the delivered product/mixture (dimensionless; wt%/100). (Enviro Tech Chemical Services, Inc., n.d.; Aubeuf‑Prieur et al., 2021).
- $w_{a}$= mass fraction of the **parent acid** (AA for PAA products; FA for PFA generator output) (dimensionless). (Enviro Tech Chemical Services, Inc., n.d.; Aubeuf‑Prieur et al., 2021).
- $w_{h}$= mass fraction of **hydrogen peroxide** in the delivered product/mixture (dimensionless). (Enviro Tech Chemical Services, Inc., n.d.; Solvay Chemicals, Inc., 2003).
- $COD_{ThOD}$= ThOD‑based oxygen‑demand contribution **per mg active peracid delivered** (mg O_2_/mg active peracid).
- $COD_{app}$= **apparent dichromate COD** contribution **per mg active peracid delivered**, defined as $COD_{ThOD}$plus an H_2_O_2_ interference term (mg O_2_/mg active peracid). (Kang et al., 1999).
- $COD_{p}$= stoichiometric oxygen‑demand factor of the **peracid component** (mg O_2_/mg component): PAA = 0.63, PFA = 0.00 (Table 6).
- $COD_{a}$= stoichiometric oxygen‑demand factor of the **parent acid component** (mg O_2_/mg component): AA = 1.07, FA = 0.35 (Table 6).
- 0.4706 = **COD/** H_2_O_2_ interference ratio (mg O_2_ apparent COD per mg H_2_O_2_) used for dichromate COD bias correction (Kang et al., 1999)

1. **Assumptions & calculation framework (used for Table S5)**

**A) COD factors (consistent with Table 6)**

- Peracetic acid (PAA): 0.63 mg O₂/mg
- Acetic acid (AA): 1.07 mg O₂/mg
- Performic acid (PFA): 0.00 mg O₂/mg
- Formic acid (FA): 0.35 mg O₂/mg
- H₂O₂: 0.4706 mg O₂/mg as apparent COD due to dichromate COD interference (not carbonaceous oxygen demand).

**B) “Per mg active peracid delivered” scaling**

For each product, the oxygen-demand contribution per mg active peracid is:

ThOD-only (carbonaceous oxygen demand):

COD_ThOD_​=COD_p_​+COD_a_​(w_a_/w_p_​​)

Apparent dichromate COD (includes H₂O₂ interference term):

COD_app_​=COD_ThOD_​+0.4706(w_h_/w_p_​)

**C) PAA “real product” mass fractions used**

- Peragreen® 22 TDS: PAA 21.8–22.8%, H₂O₂ 4.7–5.2%, AA 40–50% → we use low-COD end (max PAA, min AA, min H₂O₂) and high-COD end (min PAA, max AA, max H₂O₂).
- Peragreen® 15 TDS: PAA 14.8–15.7%, H₂O₂ 21.5–22.5%, AA 15.8–16.6% → same low/high approach.
- VigorOx® WWT II digest: 15% PAA / 23% H₂O₂ / 16% AA (fixed).
- Proxitane® WW-12 MSDS: 12% PAA / 18.5% H₂O₂ / 20% AA (fixed).

**D) PFA bounding (10–16 wt% active PFA)**

Because generator output composition (residual FA/H₂O₂) is not standardized in public specs, PFA carryover is bound using a stoichiometric equilibrium framework constrained by published information:

- Active PFA in mixture: 10–16 wt% (Consistent with Kemira disclosures).
- Precursors: formic acid solutions ~70–80 wt%, hydrogen peroxide solutions ~30–50 wt% (reported as typical precursor strengths in Kemira patent disclosure).
- Assuming 1:1 molar feed FA:H₂O₂ (neutral assumption when only “constant ratio” is disclosed).

From these constraints, the implied conversion fraction $f$is bounded at:

- $f\approx0.202$to $0.462$(dimensionless)

This yields PFA oxygen-demand bounds per mg active PFA delivered:

- PFA COD_ThOD_: 0.302–1.023 mg O₂/mg active PFA
- PFA COD_app_: 0.603–2.040 mg O₂/mg active PFA
  (using the interference factor from Kang et al.).

**How ratio bounds are computed:**
For each PAA case, we compute ratio ranges as:

- **Minimum ratio** = $COD_{PAA}/COD_{PFA,\text{ }max}$(worst-case PFA carryover → highest PFA COD → smallest ratio)
- **Maximum ratio** = $COD_{PAA}/COD_{PFA,\text{ }min}$(best-case PFA carryover → lowest PFA COD → largest ratio)

Table S4. PAA compositions are taken from Peragreen® 22 and Peragreen® 15 technical data sheets (Enviro Tech Chemical Services, Inc., n.d.), the VigorOx® WWT II formulation description (PeroxyChem, 2014), and the Proxitane® WW‑12 MSDS (Solvay Chemicals, Inc., 2003). PFA bounds use the disclosed 10–16 wt% active-PFA range and the presence of unreacted FA and H2O2 in the equilibrium mixture (Aubeuf‑Prieur et al., 2021).The H2O2 interference coefficient (0.4706) follows the dichromate COD interference correction described by Kang et al. (1999). Sources for PAA cases: Peragreen 22 TDS ranges, Peragreen 15 TDS ranges, VigorOx WWT II digest, Proxitane WW-12 MSDS

| **PAA product case** | $w_{p}$**(PAA)** | $w_{AA}$ | $w_{H2O2}$ | **COD_ThOD (mg O₂ / mg active PAA)** | **COD_app (mg O₂ / mg active PAA)** | **PAA/PFA ratio (ThOD) min** | **PAA/PFA ratio (ThOD) max** | **PAA/PFA ratio (apparent COD) min** | **PAA/PFA ratio (apparent COD) max** |
| --- | --- | --- | --- | --- | --- | --- | --- | --- | --- |
| **PAA-22 (Peragreen 22) low-COD end** | 0.228 | 0.400 | 0.047 | 2.507 | 2.604 | 2.45 | 8.29 | 1.28 | 4.32 |
| **PAA-22 (Peragreen 22) high-COD end** | 0.218 | 0.500 | 0.052 | 3.084 | 3.196 | 3.01 | 10.20 | 1.57 | 5.30 |
| **PAA-15 (Peragreen 15) low-COD end** | 0.157 | 0.158 | 0.215 | 1.707 | 2.351 | 1.67 | 5.65 | 1.15 | 3.90 |
| **PAA-15 (Peragreen 15) high-COD end** | 0.148 | 0.166 | 0.225 | 1.830 | 2.546 | 1.79 | 6.05 | 1.25 | 4.22 |
| PAA-15/23/16 (VigorOx WWT II) | 0.150 | 0.160 | 0.230 | 1.771 | 2.493 | 1.73 | 5.86 | 1.22 | 4.14 |
| PAA-12/18.5/20 (Proxitane WW-12 MSDS) | 0.120 | 0.200 | 0.185 | 2.413 | 3.139 | 2.36 | 7.98 | 1.54 | 5.21 |

Notes:

- “min” and “max” ratio columns are computed by dividing the PAA oxygen‑demand metric by the upper and lower bounds, respectively, of the corresponding PFA metric under the bounding framework (Aubeuf‑Prieur et al., 2021; Kang et al., 1999).
- “PAA/PFA ratio” > 1 means more oxygen-demand contribution per mg active for PAA than PFA.

Two ratio types are shown:

(i) ThOD-based (carbonaceous oxygen demand), and

(ii) Apparent dichromate COD (includes possible H₂O₂ measurement interference).

1. Summary ranges (across the PAA products listed; PFA bounded at 10–16 wt% active)

- **ThOD-based PAA/PFA ratio range:** **~1.67× to ~10.20×** (environmental oxygen-demand contribution emphasis
- **Apparent dichromate COD ratio range:** **~1.15× to ~5.30×** (includes potential COD-test inflation from residual peroxide)

1. Disinfectant Selection Decision Tree

The decision tree diagram in Figure S7 is intended to provide a screening-level decision framework that translates the single-site, bench-scale findings of this study (ammonia-rich secondary effluent) into a practical selection logic for sodium hypochlorite-derived chloramines (NaOCl), PFA, and PAA. The tree is intended to support local validation (pilot or full-scale) prior to adoption because conclusions are based on one facility and were not verified across multiple sites or full-scale operating conditions. Decision nodes reflect key operational conditions: high NH₃-N (chloramines likely), whether a persistent residual is required (e.g., storage/distribution), whether broad (non-selective) control of ARB and non‑ARB is the target, and whether PFA implementation is feasible (e.g., on-site generation and associated capital/operational constraints). Output boxes summarize study-aligned options: PFA is indicated when feasible and when durable control is prioritized, using the study-derived durability region (ICT ≥ 28–34 mg·min/L) where recovery was minimal under extended incubation/RI; PAA is indicated when residual is not required and additional ICT validation is planned because recovery persisted within the tested ICT range despite observed ARB benefits; SH/chloramines is indicated when residual is required, with CAPEX/OPEX cues (e.g., basin volume, Disinfection byproduct (DBP)/dechlorination burden) included as general engineering considerations rather than study-quantified outcomes.

**
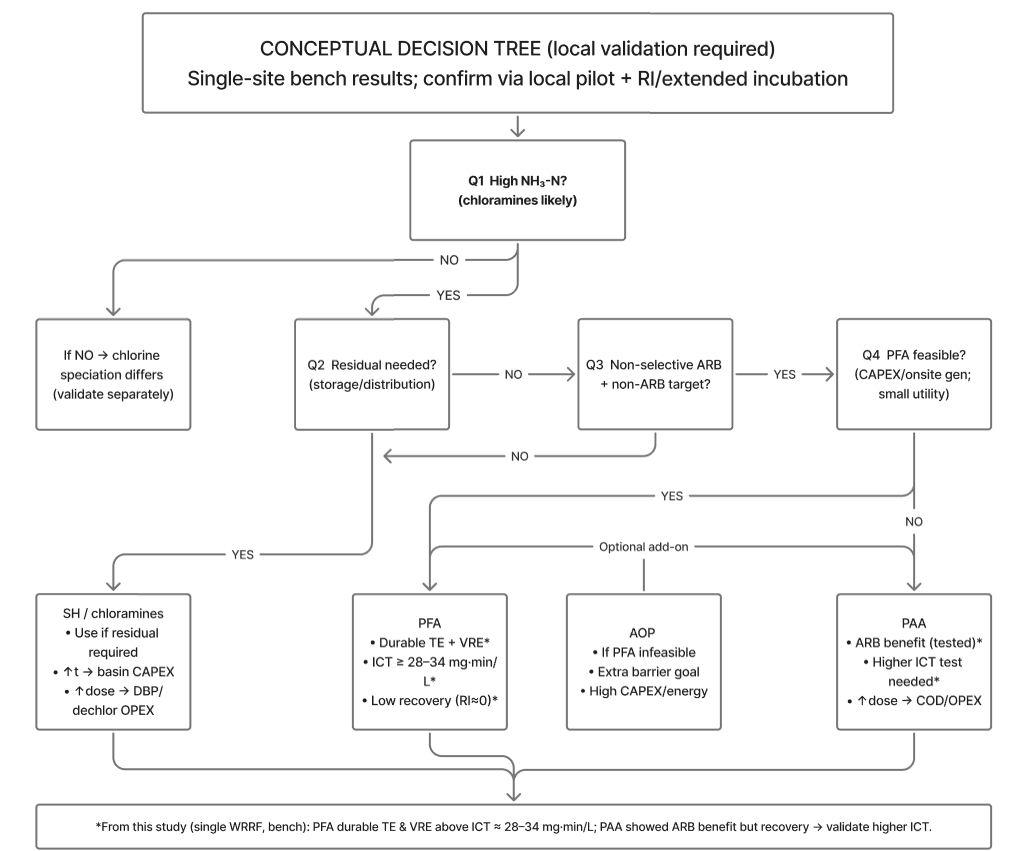
**

Figure S7. Conceptual operational decision tree for disinfectant selection using ICT–reactivation evidence.

**Note**: Cost/DBP/COD-related cues in the figure are included only to support feasibility screening and should be evaluated using site-specific design constraints and regulatory context during local validation. Cost and DBP factors are conceptual placeholders outside the scope of this study.

**Bibliography**

Aubeuf‑Prieur, P., Porat, I., Stammegna, M., & Greus, S. (2021). Performic acid production systems and methods (WO2021183516A1). World Intellectual Property Organization. <https://patents.google.com/patent/WO2021183516A1/en>

Akiyama, T., & Savin, M. C. (2010). Populations of antibiotic-resistant coliform bacteria change rapidly in a wastewater effluent dominated stream. Science of the Total Environment, 408(24), 6192–6201. <https://doi.org/10.1016/j.scitotenv.2010.08.055>

Bagagnan, S., Jusselme, M. D., Alphonse, V., Guerin-Rechdaoui, S., Marconi, A., Rocher, V., & Moilleron, R. (2024). Assessing the effectiveness of performic acid disinfection on effluents: Focusing on bacterial abundance and diversity. Environmental Science and Pollution Research, 31, 58252–58262. <https://doi.org/10.1007/s11356-024-34958-4>

Campo, N., De Flora, C., Maffettone, R., Manoli, K., Sarathy, S., Santoro, D., … Auset, M. (2020). Inactivation kinetics of antibiotic resistant Escherichia coli in secondary wastewater effluents by peracetic and performic acids. Water Research, 169, 115227. <https://doi.org/10.1016/j.watres.2019.115227>

Ding, N., Liu, K., Jiang, L., & Liu, H. (2023). The temperature-dependent kinetics and bacteria regrowth by performic acid and sodium hypochlorite disinfection. Water Science & Technology, 88(9), 2233–2245. <https://doi.org/10.2166/wst.2023.351>

Enviro Tech Chemical Services, Inc. (n.d.). Peragreen® 22 technical data sheet (Product #69) [PDF]. <https://envirotech.com/wp-content/uploads/_pda/069-peragreen-22-TDS.pdf>

Enviro Tech Chemical Services, Inc. (n.d.). Peragreen® 15% technical data sheet (Product #63) [PDF]. <https://envirotech.com/wp-content/uploads/_pda/063-peragreen-15-TDS.pdf>

Gehr, R., Chen, D., & Moreau, M. (2009). Performic acid (PFA): tests on an advanced primary effluent show promising disinfection performance. Water Science & Technology, 59(1), 89–96. <https://doi.org/10.2166/wst.2009.761>

Hornsby, G., Ibitoye, T. D., Keelara, S., & Harris, A. (2023). Validation of a modified IDEXX defined-substrate assay for detection of antimicrobial resistant E. coli in environmental reservoirs. Environmental Science: Processes & Impacts, 25, 37–43. <https://doi.org/10.1039/D2EM00189F>

Jimenez, K., Kong, Y., Zhang, Y., Ferketic, D., Nagori, S. K., Yang, J., … Jay, J. A. (2025). Evaluation of a modified IDEXX method for antimicrobial resistance monitoring of extended beta-lactamases-producing Escherichia coli in impacted waters near the U.S.-Mexico border. One Health, 20, 100997. https://doi.org/10.1016/j.onehlt.2025.100997

Kang, Y. W., Cho, M.-J., & Hwang, K.-Y. (1999). Correction of hydrogen peroxide interference on standard chemical oxygen demand test. Water Research, 33(5), 1247–1251. <https://doi.org/10.1016/S0043-1354(98)00315-7>

Manoli, K., Bell, K. Y., Jang, E., Norton, J. W., Mitobe, S., Parra, A., … Santoro, D. (2024). Reliable wastewater disinfection with peracids: Integrating batch kinetics and hydrodynamics. Proceedings of the Water Environment Federation (WEFTEC 2024 Conference Paper). <https://doi.org/10.2175/193864718825159567>

Nyangaresi, P. O., Manoli, K., Norton, J., Bell, K. Y., Zhang, C. S., Da Silva, A. K., & Beck, S. E. (2026). Performic acid disinfection in wastewater effluent and combined sewer overflows: A review. *Water Research*, *299*, 125837. https://doi.org/10.1016/j.watres.2026.125837

Ocampo-Rodríguez, D. B., Vázquez-Rodríguez, G. A., Rodríguez, J. A., González Sandoval, M. d. R., Iturbe-Acosta, U., Martínez Hernández, S., & Coronel-Olivares, C. (2023). Kinetic models of disinfection with sodium hypochlorite and peracetic acid of bacteria isolated from the effluent of a WWTP. Water, 15(11), 2019. <https://doi.org/10.3390/w15112019>

PeroxyChem. (2014, April). An introduction to VigorOx® WWT II for wastewater disinfection (Disinfection Digest, Issue 1) [PDF]. <https://www.microbialcontrol.fmc.com/media/97706/apr2014_introductiontovigorox02_01_wwt_14.pdf>

Ragazzo, P., Chiucchini, N., Piccolo, V., Spadolini, M., Carrer, S., Zanon, F., & Gehr, R. (2020). Wastewater disinfection: Long-term laboratory and full-scale studies on performic acid in comparison with peracetic acid and chlorine. Water Research, 184, 116169. <https://doi.org/10.1016/j.watres.2020.116169>

Sahulka, S. Q., Bhattarai, B., Bhattacharjee, A. S., Tanner, W., Mahar, R. B., & Goel, R. (2021). Differences in chlorine and peracetic acid disinfection kinetics of Enterococcus faecalis and Escherichia fergusonii and their susceptible strains based on gene expressions and genomics. Water Research, 203, 117480. <https://doi.org/10.1016/j.watres.2021.117480>

Solvay Chemicals, Inc. (2003, November 10). Proxitane® WW‑12 Microbiocide: Material Safety Data Sheet (MSDS No. WW12‑1103). <https://assets.greenbook.net/14-39-56-06-02-2017-Proxitane_WW_12_Microbiocide_10_12_2004_3_39_52_PM.pdf>

U.S. Environmental Protection Agency. (2007). Guidelines establishing test procedures for the analysis of pollutants; Analytical methods for the biological pollutants in wastewater and sewage sludge; Final rule. Federal Register, 72(57), 14220–14243.
